# Nanosecond Pulsed Electric Field Only Transiently Affects the Cellular and Molecular Processes of Leydig Cells

**DOI:** 10.3390/ijms222011236

**Published:** 2021-10-18

**Authors:** Wiktoria Kasprzycka, Alicja Trębińska-Stryjewska, Rafał Bogdan Lewandowski, Małgorzata Stępińska, Paulina Natalia Osuchowska, Monika Dobrzyńska, Yahia Achour, Łukasz Paweł Osuchowski, Jacek Starzyński, Zygmunt Mierczyk, Elżbieta Anna Trafny

**Affiliations:** 1 Biomedical Engineering Centre, Institute of Optoelectronics, Military University of Technology, 00-908 Warsaw, Poland; wiktoria.kasprzycka@wat.edu.pl (W.K.); alicja.trebinska@wat.edu.pl (A.T.-S.); rafal.lewandowski@wat.edu.pl (R.B.L.); malgorzata.stepinska@wat.edu.pl (M.S.); paulina.osuchowska@wat.edu.pl (P.N.O.); monika.dobrzynska@wat.edu.pl (M.D.); lukasz.osuchowski@wat.edu.pl (Ł.P.O.); zygmunt.mierczyk@wat.edu.pl (Z.M.); 2 Faculty of Electronics, Military University of Technology, 00-908 Warsaw, Poland; yahiaaachour@gmail.com (Y.A.); jstar@ee.pw.edu.pl (J.S.)

**Keywords:** nanosecond pulsed electric field, Leydig TM3 cells, microvilli, adhesion, expression microarrays

## Abstract

The purpose of this study was to verify whether the nanosecond pulsed electric field, not eliciting thermal effects, permanently changes the molecular processes and gene expression of Leydig TM3 cells. The cells were exposed to a moderate electric field (80 quasi-rectangular shape pulses, 60 ns pulse width, and an electric field of 14 kV/cm). The putative disturbances were recorded over 24 h. After exposure to the nanosecond pulsed electric field, a 19% increase in cell diameter, a loss of microvilli, and a 70% reduction in cell adhesion were observed. Some cells showed the nonapoptotic externalization of phosphatidylserine through the pores in the plasma membrane. The cell proportion in the subG1 phase increased by 8% at the expense of the S and G2/M phases, and the DNA was fragmented in a small proportion of the cells. The membrane mitochondrial potential and superoxide content decreased by 37% and 23%, respectively. Microarray’s transcriptome analysis demonstrated a negative transient effect on the expression of genes involved in oxidative phosphorylation, DNA repair, cell proliferation, and the overexpression of plasma membrane proteins. We conclude that nanosecond pulsed electric field affected the physiology and gene expression of TM3 cells transiently, with a noticeable heterogeneity of cellular responses.

## 1. Introduction

The impact of the nanosecond pulsed electric field (nsPEF) on mammalian cell physiology has been studied for over two decades, producing some fundamental observations and providing a basis for this technology’s practical use. In general, the effects of the pulsed electric field can be reversible without additional thermal effects (gene electrotransfer or electrochemotherapy), or irreversible with or without thermal effects (ablation of neoplastic tumours) [1,2]. Electroporation based on milli- or microsecond pulses is a valued technique used in genetic engineering and gene expression regulation [3]. Research shows that high-voltage nanosecond pulses can also be an effective tool for transporting various particles into the cells, especially those with a charge, e.g., nucleic acids [4,5]. The short duration of these pulses can prevent the thermal effects that otherwise might adversely affect the cells. However, the relatively smaller pore size in the plasma membrane, due to nsPEF exposure, might result in low efficiency in the delivery of large DNA molecules (i.e., plasmids) into cells [5]. A nanosecond pulsed electric field may be more suitable for transferring small RNA species through the plasma membrane [6]. Thus, it is essential to establish the conditions of nsPEF exposure that lead to the nanoelectroporation of cellular membranes with the least amount of detrimental effects on the cells.

Among the effects of nsPEF, cell swelling and blebbing, the activation of protein kinase pathways, cell cycle arrest in the G2/M phase and, above all, electroporation of the plasma and intracellular membranes of organelles leading to apoptosis or necrosis, have already been documented [7,8,9,10]. Various mammalian cells in vitro showed different levels of sensitivity to the treatment [11,12]. The factors that contribute to cell susceptibility to nsPEF-induced damage or, on the contrary, allow them to survive, remain an open question.

With regard to the already known cell injury mechanisms by nsPEF observed for the other cell lines, we chose Leydig cells (TM3) as the object of this study. The reasons behind this decision were as follows: (i) TM3 cells proliferate at a high rate (divide every 16 h) [13] and, thus, the damages are quickly observable; (ii) TM3 cells have numerous microvilli [14], a membrane reserve [15], that can be used for the rapid repair of the cytoplasmic membrane, which is a primary target of nsPEF; (iii) TM3 cells also have abundant mitochondria [16] and, hence, any ATP losses caused by the outflow of molecules from the cell through the nanopores due to exposure to nsPEF can be replaced relatively quickly.

Testing the sensitivity of Leydig cells to nsPEF has some practical implications. Leydig cells secrete more than 95% of the testosterone produced in the body. This hormone is responsible for the production of sperm and influences the adipose tissue, muscle, and cardiovascular system in men. The STAR protein is responsible for the transport of cholesterol, a substrate for testosterone synthesis, to the mitochondria. Regulation of *STAR* expression can be mediated by miRNA [17,18], with the effect of increased or decreased expression, using miR-150 antagomir or miR-150 agomir. Furthermore, the viability of Leydig cells can be affected by external factors, such as long-term cigarette smoking. This could be prevented by introducing an miR-138-5p mimic into the cells, thus inhibiting TM3 Leydig cell apoptosis caused by exposure to cigarette smoke [19]. The ability to regulate the activity and survival of Leydig cells by introducing small interfering RNAs upon nsPEF exposure could provide the means to influence male testis and fertility, providing no damage to the cells.

In 2013, Moen et al. [20] formulated a hypothesis on the so-called stressors that are the causes of unfavourable stimuli on the cell upon exposure to nsPEF. Therefore, we decided to concurrently carry out several analyses to reveal the possible stressors after the exposure of TM3 cells to nsPEF. In the search for these stressors, we monitored the plasma membrane’s continuity, mitochondrial membrane potential as a marker of the homeostasis damage, cell swelling and blebbing, and reactive oxygen species (ROS) levels in the cytoplasm and mitochondria. Moreover, the cell morphology, variations in cell adhesion, and the cytoskeleton were studied. Simultaneously, we looked at the genotoxic DNA damage and perturbations in the cell cycle phases as inextricably linked phenomena. The analyses mentioned above were accompanied by the search for genome-wide expression changes in TM3 cells after exposure to nsPEF at three different time points, which has not been analysed so far.

Our methodological approach demonstrated that all profound changes in cell homeostasis observed immediately after exposure to nsPEF had only a transient character, and TM3 cells did not suffer much from such a treatment. This opens up the opportunity for using the nsPEF parameters for research on sRNA electrotransfer, or other applications of the reversible electroporation of TM3 cells.

## 2. Results

### 2.1. Temperature Not Elevated during nsPEF Treatment

The nsPEF treatment comprised 80 pulses, each pulse was 60 ns in length, at a 14 kV/cm electric field (see Methods section for details). The TM3 cells were exposed in suspension. Sham-treated cells were analysed alongside the exposed cells to control the effects of cell harvesting and preparation. Detailed descriptions of the sample preparation for exposure and analysis can be found in the Materials and Methods section. To exclude the impact of the temperature increases on the TM3 cells upon exposure to nsPEF, we carefully monitored this parameter during the nsPEF treatment. As shown in Supplementary Figure S1d, the overall increase in the sample’s temperature during the treatment period did not exceed 0.2 °C; therefore, this stressor was excluded from further analysis. 

### 2.2. A Fraction of Cells Increased in Size, and Their Morphology Temporarily Changed 

**Figure 1:**
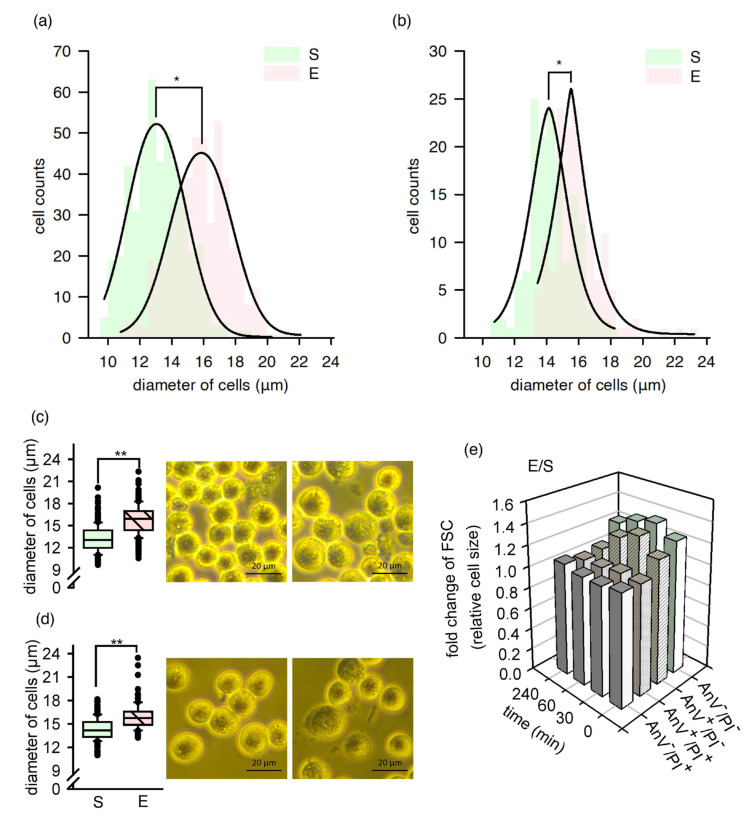
The increase in the size of the TM3 cells due to nsPEF treatment. S: sham-exposed cells. E: nsPEF-exposed cells. (**a**) Cell diameter distribution within the samples S and E in three independent experiments, with a total of 450 cells scored for each type of sample. The data were collected 5 min after the treatment. (**b**) Cell diameter distribution within the samples S and E in one independent experiment, with 150 cells scored for each sample. The data were collected 240 min after the treatment. (**a**,**b**) The solid line shows the Gaussian regression for the given data. (**c**,**d**) The cells’ diameter in samples S and E displayed as box plots (a median value, box: 25th and 75th percentiles (bottom and top of the boxes). Whiskers: 5th and 95th percentiles) together with the corresponding images of cells recorded using the inverted optical microscope. The data corresponds to plots (**a**,**b**), respectively. (**e**) The fold change of forward scatter (FSC) (relative size) of nsPEF-exposed cells after the Annexin V-Alexa Fluor 488 and propidium iodide staining compared to the sham-exposed cells stained as above. The FSC signals were recorded at 0, 30, 60, and 240 min after nsPEF exposure with flow cytometry. The independent experiments were performed five (at 0 min), three (at 30 min), and two (at 60 and 240 min) times. Standard deviations for the values in the graph have been presented separately (Table S1) to ensure data legibility. The asterisk represents the significant difference: * *p* < 0.0001 in the Kolmogorov–Smirnov test, and ** *p* < 0.001 in the Student’s *t*-test, between the sham and exposed samples.

A significant increase in cell diameter was observed under an inverted light microscope immediately after nsPEF exposure. The distribution of the cell diameters differed significantly between the sham and exposed samples at 5 min after exposure (Figure 1a). After 240 min, both distributions showed more overlap, but the difference was still significant (Figure 1b). The mean diameter of the cell in the sham samples was 13.2 ± 1.7 μm, in comparison to 15.8 ± 1.9 μm for the exposed cells at 5 min after treatment (Figure 1c). After 240 min, the values were equal to 14.3 ± 1.4 μm and 15.9 ± 1.6 μm, respectively (Figure 1d). 

Confirming the above findings, the relative cell size was monitored with flow cytometry at 0, 30, 60, and 240 min after nsPEF exposure, and recorded as a fold change of the FSC (forward scatter) values relative to the sham. The level of FSC signals varies in accordance with the following cell size [21]. Cells were additionally stained and gated on propidium iodide (PI) versus Alexa Fluor 488 (AnV) graphs. After nsPEF exposure, only PI negative cells showed an increase in their relative size (Figure 1e). The highest difference between the exposed and sham samples was observed at 30 min after exposure, and then the FSC fold-change values decreased. At 240 min, the relative size of AnV^+^/PI^−^ cells exposed to nsPEF was comparable to those in the sham samples. However, the cell volume of AnV^−^/PI^−^ cells in the exposed samples did not decrease to the initial values. These results point to a heterogeneous response of the TM3 cells to nsPEF exposure, with cellular swelling observed only for specific subpopulations, and dependent on the cell’s physiological state. Moreover, some of the observed changes were reversible.

While examining cell morphology with SEM, the cells should be attached to a surface and dehydrated. During the procedure’s subsequent steps, damaged nonadherent cells can be washed away and omitted from observations. In our study, we filtered cell suspensions through a polymer membrane with a pore size of 8 μm. This innovative approach allowed for preserving the entire population and reduced the washout of dead and poorly adhesive cells.

**Figure 2:**
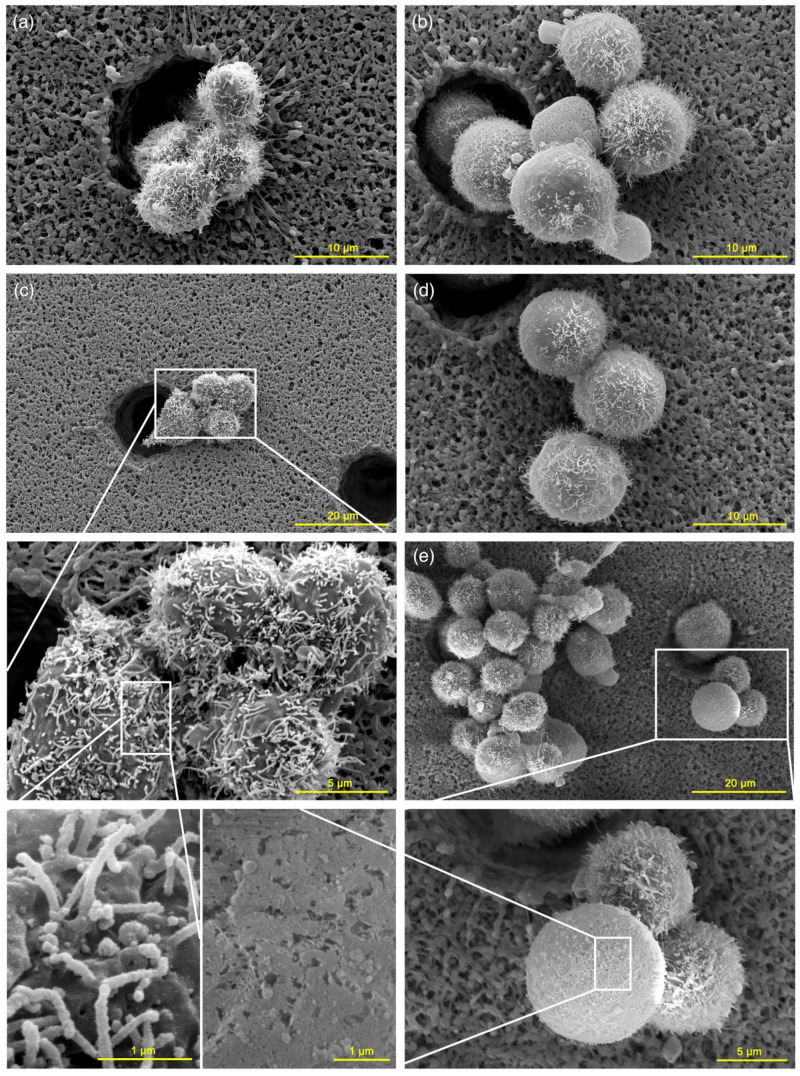
Cell morphology. SEM images. The cells were filtered through the polymer membrane and fixed with paraformaldehyde immediately after nsPEF exposure. (**a**,**c**) Sham samples. Morphology of TM3 cells in control samples with a folded cell membrane and numerous microvilli on the surface. (**b**,**d**,**e**) nsPEF-exposed cells. An increase in the diameter of cells, loss of microvilli, areas of roughness, and plasma membrane blebbing can be observed.

The increase of cell size immediately after nsPEF exposure was also observed under SEM, consistent with the light microscopy and flow cytometry results. The differences in cell sizes are visible in Figure 2, where sham (Figure 2a) and exposed cells (Figure 2b) are shown with the same magnification. Besides these changes, some morphological modifications caused by nsPEF could also be observed. Some of the exposed cells had a reduced number of microvilli (Figure 2b,d–e), while others showed membrane damage manifested by rough areas on the cell surface (Figure 2e). Cells might respond to membrane permeabilization by producing plasma membrane vesicles as a mechanism of preventing cell injury. This phenomenon of so-called “blebbing” was observed in the exposed cells fixed immediately postexposure (Figure 2b). These changes are not visible in the sham-treated cells. A loss of microvilli and cell morphology changes were characteristic features of nsPEF-exposed TM3 cells.

### 2.3. Adhesion of nsPEF-Exposed Cells Diminished 

**Figure 3:**
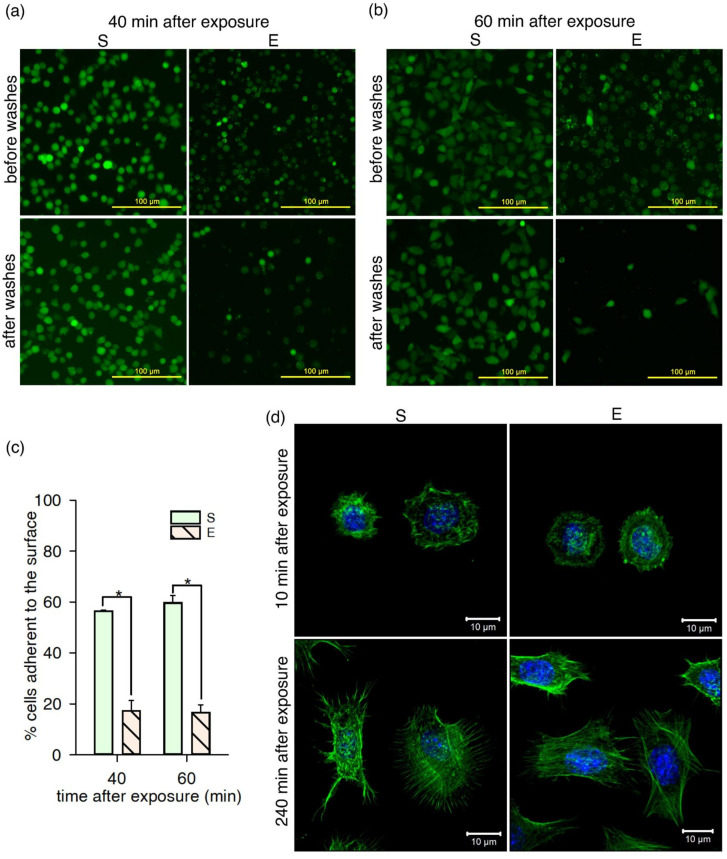
Adhesion and actin cytoskeleton of TM3 cells. S: sham-exposed cells. E: nsPEF-exposed cells. (**a**,**b**) Wash assay: TM3 cells were stained with calcein-AM before nsPEF exposure, seeded on the plastic surface, and visualized by epifluorescence microscopy before and after washes. The wash assay was carried out at 40 (**a**) and 60 min (**b**) after nsPEF exposure. Healthy intact cells were observed without the exposure (S samples, left panels), and two populations were visible after nsPEF exposure (E samples, right panels): cells with high calcein fluorescence intensity that attached to the surface, and cells with a diminished calcein staining that were mostly washed out. The yellow bar represents 100 μm. (**c**) Differences in cell attachment between sham- and nsPEF-exposed samples were detected in the wash assay at 40 min and 60 min after treatment. The bars represent the percentage of cells that adhered to the surface (mean value ± SD of three biological replicates, each in three technical repetitions). Asterisk represents the significant difference between the fractions of cells adhering to the surface in sham- and nsPEF-exposed samples at 40 and 60 min (* *p* < 0.001, Student’s *t*-test). (**d**) Actin cytoskeleton stained with phalloidin-Alexa Fluor 488 (green), and the nucleus stained with Hoechst (blue). The cells were fixed after 10 min (upper row), and 240 min (lower row), post-treatment, and visualized under CLSM. The white bar represents 10 µm.

Changes in the plasma membrane could impact the adhesion efficiency of TM3 cells. To verify this hypothesis, the efficiency of cell attachment to a polystyrene surface in a serum-free medium was determined in a wash assay, at 40 and 60 min after exposure to nsPEF. The cells were stained with calcein before the treatment, and the medium was depleted of serum. There was a significant difference (Student’s *t*-test, *p* < 0.001) between a fraction of the cells adhering to the surfaces in the sham and exposed samples at both time points (Figure 3c). Sham-treated cells at 40 and 60 min were attached to the surface at a higher percentage (56 ± 0.41% and 60 ± 3.0%, respectively) than nsPEF-exposed cells (17 ± 4.1% and 17 ± 3.1%, respectively). At 40 min after exposure, the cells were rounded with a uniform calcein staining in the sham sample cells, and a less intense mostly patched staining of the exposed cells (Figure 3a). After another 20 min, the cells in the sham samples flattened and adhered to the surface (Figure 3b, left column). In the nsPEF-exposed samples, only cells with uniform calcein staining were attached to the surface and showed signs of spreading; the round cells with patched staining were washed out (Figure 3b, right column). These findings indicate that, in serum-free conditions, nsPEF treatment impairs the attachment process of the affected TM3 cell subpopulation.

The decreased adhesion might suggest cell cytoskeleton changes but, in this study, no significant changes in the staining of actin filaments in the cytoplasm were revealed when comparing the exposed and sham samples (Figure 3d). However, only the formaldehyde-fixed adherent cells were observed in these experiments under the confocal microscope. We cannot exclude the possibility that swollen cells deprived of microvilli were washed out from the preparations. This observation could suggest the microvilli-related mechanism of diminished cell adhesion. 

### 2.4. Apoptosis in Only a Small Fraction of Exposed Cells 

In order to explore further plasma membrane damage after nsPEF, we stained TM3 cells with the LIVE/DEAD™ Viability/Cytotoxicity Kit. The procedure did not require the wash steps, and the cells could be gently spun to the bottom before staining. Thus, we assumed that almost the entire cell population was retained for these experiments. By this means, the elongated and attached-to-the-surface cells were observed at 240 min post-exposure (Supplementary Figure S2). Their number did not decrease significantly compared to the sham samples. The cells with a damaged membrane (stained by ethidium homodimer-1), but with high esterase activity (calcein-stained, red dots surrounded by green colour), were visible 15 min after nsPEF treatment (Supplementary Figure S2, right column, upper row). Only cells stained with a single dye were recorded at 240 min after exposure, and only in the sham samples (Supplementary Figure S2, lower row). Overall, these results indicate that, despite some damage to the plasma membrane after nsPEF exposure, most of the cells recovered, as demonstrated by the fact that they remained attached to the surface and retained esterase activity.

**Figure 4:**
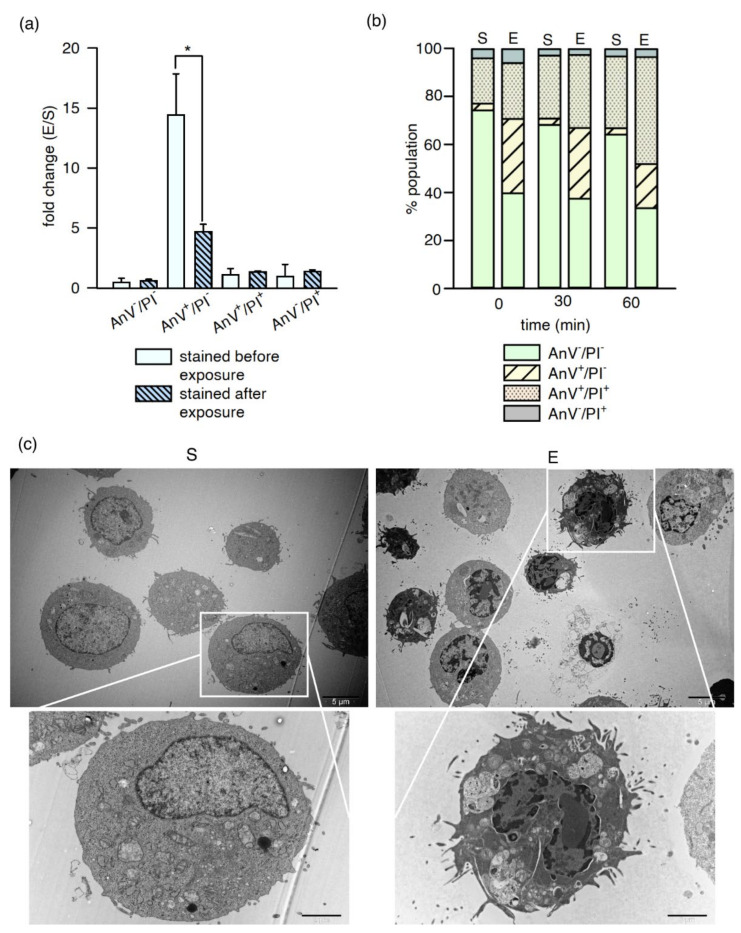
The presence of phosphatidylserine and other apoptosis-related changes in TM3 cells. S: sham-exposed cells. E: nsPEF-exposed cells. (**a**) The presence of phosphatidylserine on the membrane’s outer leaflet of cells stained with AnnexinV-Alexa Fluor 488 (AnV) and propidium iodide (PI) before the treatment with nsPEF, and immediately after treatment. Four populations of TM3 cells were distinguished by flow cytometry, and 10,000 events were recorded for each sample. The results are expressed as fold changes relative to sham-exposed samples. The bars represent median value and the 75th percentile of five (cells stained before exposure), or four (cells stained after exposure), independent experiments, with three biological repetitions per condition; the difference was significant (Student’s *t*-test (* *p* < 0.05)) for AnV^+^/PI^-^ cells. (**b**) The phosphatidylserine’s externalization at the surface of cells stained before the nsPEF treatment, as described above; the percentage of cell populations in the samples S and E during 60 min post-exposure. The mean value of five (at 0 min), three (at 30 min), or two (at 60 min) independent experiments, with three biological repetitions per condition. (**c**) TEM images of sham- and nsPEF-exposed TM3 cells. Healthy cells with intact plasma and nuclear membranes predominate in sham-exposed control samples (left column). Numerous cells with morphological signs of apoptosis, such as nuclear membrane damage, chromatin condensation, and irregular cell outline, can be observed after nsPEF treatment (right column). The black bar represents 5 and 2 µm, respectively.

Phosphatidylserine (PS) molecules may be externalized within seconds because of the appearance of nanopores in the plasma membrane induced by high-energy electric pulses. At a later time, PS can also be externalized during the course of apoptosis in response to the nsPEF treatment [22,23]. To distinguish between these two scenarios, cells were stained with FITC-Annexin V and propidium iodide in two ways. The cell suspensions were subjected to nsPEF with fluorescent dyes already introduced to the aliquots and immediately analysed by flow cytometry after exposure. Alternatively, the dyes were added following nsPEF treatment, and the analysis was carried out 30 min later. As shown in Figure 4a, the population of PI-negative cells with phosphatidylserine accessible for FITC-AnV was three times higher in the samples stained before nsPEF exposure. However, in both cases, there were more AnV^+^/PI^−^ cells in nsPEF-exposed than in sham samples. This suggests that, after the initial damage, some of the exposed cells were able to repair their plasma membranes, resulting in decreased PS accessibility. However, a fraction of nsPEF-exposed cells showed signs of further membrane damage, as indicated by increasing PI staining in time (Figure 4b). Therefore, two PS externalization mechanisms could be qualified to give the above effect, i.e., apoptosis or nanoporation of the plasma membrane.

Cell images under TEM allowed for examining the putative changes occurring in the plasma membrane and organelles after exposure to nsPEF. Figure 4c shows TM3 cells in the sham (left) and exposed samples (right). Many characteristic features of the cells in different stages of apoptosis could be observed in the exposed cells. The cells showed prominent cell features in late apoptosis, i.e., karyorrhexis—nucleus fragmentation (the space between inner and outer nuclear membranes), vacuolation, and cytoplasmic segregation. Some cells also displayed more subtle changes, i.e., chromatin condensation and margination, associated with DNA breakage and the earlier stages of apoptosis. A few necrotic features in the cells after exposure could also be observed. The cell undergoing necrosis, with membrane defragmentation, organelle breakdown, and vacuolation of the cytoplasm, can be observed in the image’s lower-left corner (Figure 4c, right column). In sham-exposed cells, the caveolae (the plasma membrane invaginations), and microvilli (the plasma membrane protrusions), were also visible under TEM, in contrast to the nsPEF-exposed cells (Supplementary Figure S3).

### 2.5. Membrane Perturbations Affected the Organelles and Metabolism 

**Figure 5:**
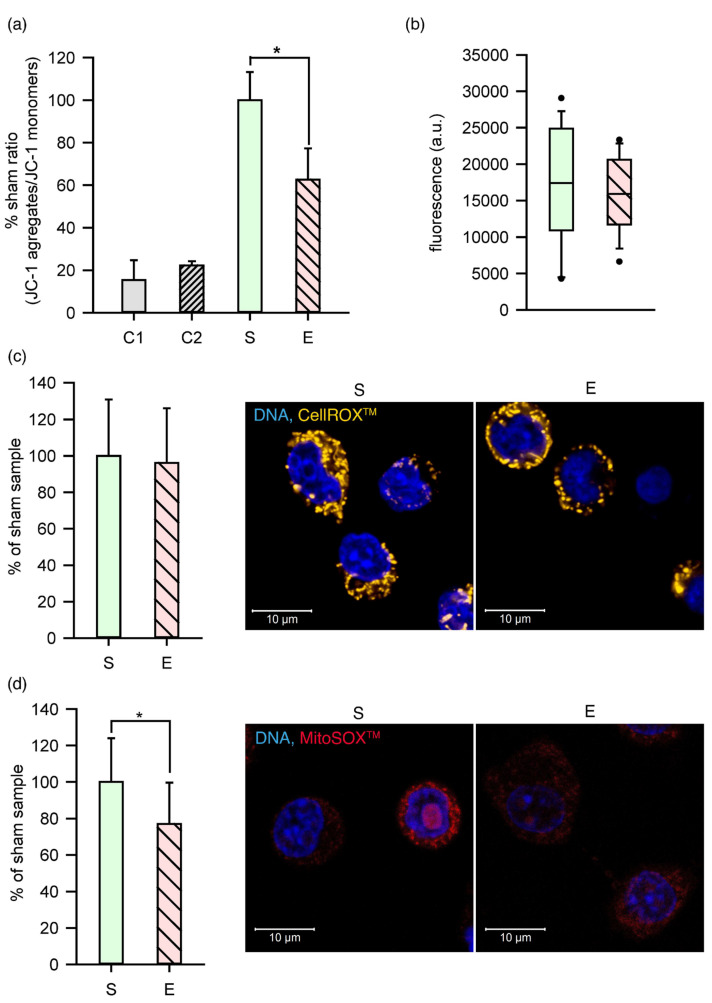
Redox balance, metabolic activity, and reactive oxygen species production in TM3 cells. S: sham-exposed cells. E: nsPEF-exposed cells. (**a**) The mitochondrial membrane potential was assayed with a JC-1 probe and recorded with flow cytometry. C1: positive control with valinomycin. C2: positive control with CCCP. Each bar shows the mean (±SD) of four independent experiments, with at least two biological repetitions. (**b**) TM3 metabolic activity measured with a PrestoBlue^®^ assay. The boxes show the median value of four independent experiments, with at least three biological and six technical repetitions per condition for each experiment (*n* = 68). The 25th and 75th percentiles are indicated by the bottoms and tops of the boxes, respectively. Whiskers: 5th and 95th percentiles. (**c**) Left: Formation of reactive oxygen species determined with the CellROX™ Orange Reagent, presented as a percentage of the average fluorescence values for S samples (±SD). Right: Images of cells under CLSM, the orange fluorescent signals are specific for reactive oxygen species detection, blue signals are from Hoechst-stained cells’ nuclei. (**d**) Left: Superoxide production by mitochondria measured with the MitoSOX™ Red Mitochondrial Superoxide, presented as a percentage of the average fluorescence values for S samples (±SD). Right: The arrangement of oxidized MitoSOX™ Red in the cells (red) and nuclei stained with Hoechst (blue). Asterisks represent the significant difference (* *p* < 0.005; the Student’s *t*-test) between the samples S and E. The white bar represents 10 µm.

The mitochondria are the organelles most sensitive to the electric field; thus, the mitochondrial membrane potential (ΔΨm) was measured as the JC-1 PE/JC-1 FITC fluorescence intensity ratio in TM3 cells and expressed as the % of the sham sample ratio (Figure 5a). The graph depicts changes in the mitochondrial membrane potential of cells after nsPEF. Two positive controls with valinomycin, the potassium selective ionophore (C1), and carbonyl cyanide m-chlorophenylhydrazone CCCP (C2), a protonophore, were used. Both compounds disconnect oxidative phosphorylation and cause the inner mitochondrial membrane potential to dissipate. ΔΨm was significantly reduced in C1 and C2 controls (respectively, 15.32 ± 9 and 22.21 ± 2.02% sham ratio) when compared to the sham control (S = 100.0 ± 13.21%). Although, to a lesser extent, depolarization of the mitochondrial membrane was also observed in the exposed cells (62.63 ± 14.64 % sham ratio). The difference was significant (Student’s *t*-test, *p* < 0.005). Loss of the mitochondrial membrane potential might evidence the increased mitochondrial membrane permeability. However, heterogeneity of the mitochondrial response to nsPEF was observed, since only 49.7% of the exposed cells, versus 32.3% of cells in the sham samples, had a diminished ΔΨm. The corresponding value in the control samples, C1 and C2, was equal to 91.0 and 85.3%, respectively.

Despite the fluctuations in the mitochondrial membrane potential, no significant difference was observed in the concentration of reactive oxygen species (ROS) in the cytoplasm of nsPEF-treated cells compared to the sham (Figure 5c). However, there was a significant difference in the superoxide levels in mitochondria (Figure 5d). The concentration of superoxide radicals was reduced by 23% in the exposed cells compared to the sham samples.

To verify the extent to which the observed changes in the cells impaired their ability to recover and proliferate, the metabolic activity of TM3 cells was assessed 24 h after nsPEF exposure. Figure 5b shows the fluorescence (a.u.), which reflected the reducing environment because of the metabolic activity of the cells in the PrestoBlue assay. No significant difference between the sham and exposed samples was observed, indicating cell recovery 24 h after nsPEF exposure.

### 2.6. DNA and the Cell Cycle Progression Disturbed

**Figure 6:**
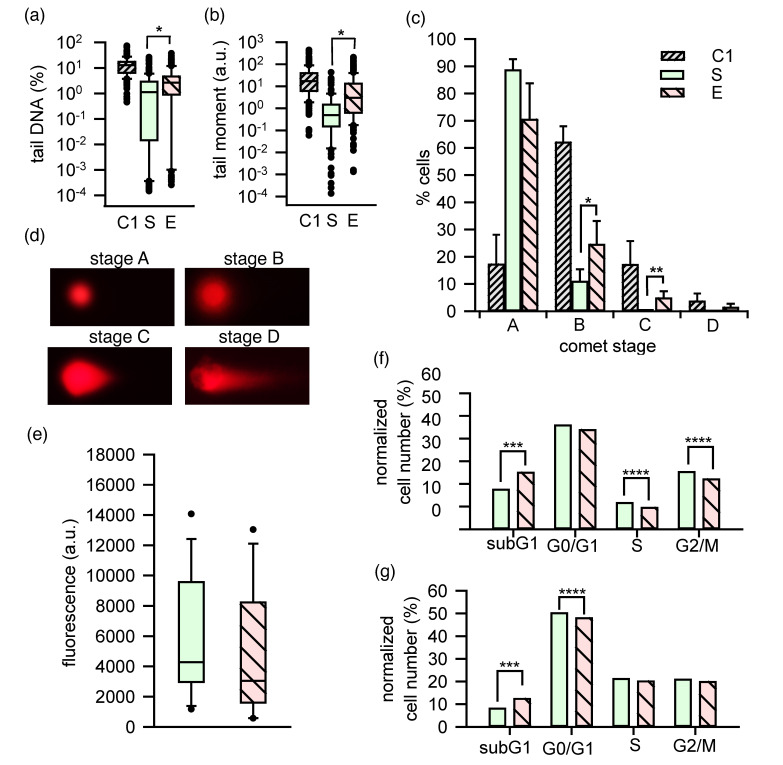
Synthesis and damage of DNA in TM3 cells. S: sham-exposed cells. E: nsPEF-exposed cells. (**a**–**d**) The extent of DNA damage was evaluated 30 min after exposure by the neutral comet assay and expressed as (**a**) tail DNA or (**b**) tail moment. C1: the cells treated with 350 µM etoposide for 24 h, positive control. Each box shows the median value (the horizontal line within a box) of four independent experiments. The 25th and 75th percentiles are indicated by the bottom and top of the boxes, respectively. Whiskers: 5th and 95th percentiles. A significant difference between sham- and nsPEF-exposed cells was determined with the nonparametric Mann–Whitney U test (* *p* < 0.001). (**c**) The percentages of cells in comet stage categories. Each bar shows the mean (±SD) of four independent experiments. Asterisks represent the significance level (* *p* < 0.001, ** *p* < 0.05, Student’s t-test) of the difference between the sham and exposed samples. (**d**) Photomicrographs of four comet stages (A–D) corresponding to nuclear DNA content in the tail observed under an epifluorescence microscope with 20 × objective lens magnification. (**e**) Synthesis of DNA by TM3 cells assessed with the CyQuant^®^ assay 24 h after exposure. The boxes show the median value of four independent experiments, with three biological replicates per condition. The 25th and 75th percentiles are indicated by the bottom and top of the boxes, respectively. Whiskers: 5th and 95th percentiles. (**f**–**g**) The cell cycle phases at 4 h (f) and 24 h. (**g**) After sham and nsPEF exposure of TM3 cells. Asterisks represent the significance level (*** *p* < 0.00001, **** *p* < 0.005, chi-square test) of the differences between the sham and exposed samples.

Because of changes in the chromatin state under TEM in our experiments, one can presume that intense checkpoint control and damage repair processes may occur in nsPEF-exposed cells. Thus, single- and double-strand breaks (SSBs and DSBs) in DNA were evaluated by the neutral comet assay within half an hour after the nsPEF stimulation. The median percentage of damaged DNA in the comet tail for the sham samples was equal to 1.11%, which was significantly lower than recorded for the exposed samples (2.65%). Simultaneously, the median percentage of tail DNA for the positive control (etoposide-treated cells) was equal to 12.36% (Figure 6a). The significant increase of the median tail moment values (*p* < 0.001) in the exposed samples, when compared to the corresponding sham samples, was also observed, and they were equal to 2.64 and 0.37 arbitrary units (a.u.), respectively; the median tail moment for the positive control was 16.71 a.u. (Figure 6b).

Photomicrographs of four comet stages (A–D) that corresponded to the nucleus DNA contents in the comet tails are shown in Figure 6d. Stage A represents cells with undamaged nuclear DNA, while stages B, C, and D represent cells with low, medium, and high levels of nuclear DNA damage, respectively. Stage E, indicating the total damage of nuclear DNA, was not observed in this study. Instead, DNA damage due to the exposure to nsPEF resulted in a significant increase in the number of cells in the B and C comet stages compared to the corresponding sham-exposed cells (Figure 6c). Additionally, the pattern of distribution of the comet stages B to D for the positive control (etoposide) and the nsPEF-exposed samples was similar.

The distribution of cell cycle phases that may be affected by the DNA damage observed in the comet assay is shown in Figure 6f–g, which depicts TM3 cell proportions at subsequent cell cycle phases in sham and exposed samples after 4 h and 24 h post nsPEF. There were significant differences between the sham- and nsPEF-exposed cells in the subG1 phase. The percentage of apoptotic cells in this phase increased from 17 to 25% at 4 h, and from 8 to 12% at 24 h, after the nsPEF treatment. Simultaneously, significant differences were found between the sham and exposed cells in the S and G2/M phases at 4 h, where the percentage of cells decreased from 12 to 9%, and from 25 to 22%, respectively. No such effect was observed 24 h after exposure to nsPEF; the significant drop in the fraction of exposed cells occurred only in the G0/G1 phase. These findings indicate a transitory negative effect of nsPEF on the proliferation of TM3 cells.

The DNA content within the sham and exposed cell populations did not significantly differ at 24 h after nsPEF exposure, as shown by the CyQuant assay (Figure 6e); however, these values tended to be lower in the exposed cells. This could indicate that most cells at 24 h after nsPEF exposure had already regenerated their metabolic pathways and started to proliferate, although some cells may have died or may not have fully recovered to homeostasis.

### 2.7. Gene Expression Largely Unchanged

**Figure 7:**
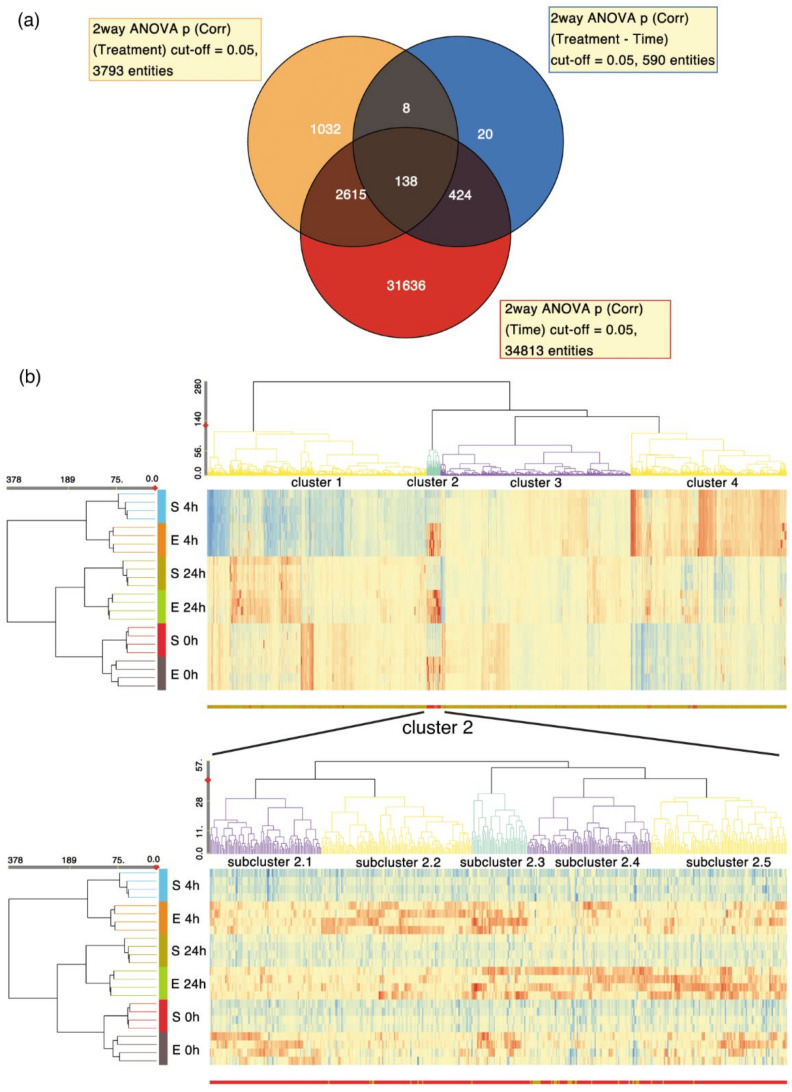
Transcriptome analysis of TM3 cells. S: sham-exposed cells. E: nsPEF-exposed cells. (**a**) Venn diagram for groups of differentially expressed genes identified by a two-way ANOVA with interaction (independent variables: treatment and time; *p* < 0.05). Orange circle: treatment-dependent differentially expressed genes. Red circle: time-dependent differentially expressed genes. Blue circle: treatment- and time-dependent differentially expressed genes. (**b**) Upper panel: unsupervised hierarchical clustering performed for all differentially expressed genes identified by ANOVA. Red colour indicates a relatively higher gene expression, and the blue, a relatively lower gene expression. Clustering on genes and conditions. Similarity measure: Euclidean. Linkage rule: Ward’s. Four main gene clusters are indicated in different colours. Lower panel: close-up of cluster 2 showing five subclusters of genes with a higher expression in nsPEF-exposed samples.

The DNA damage noticed in the comet assay could also influence gene expression. Therefore, a transcriptome analysis was performed using gene expression microarrays for the cells harvested at 0, 4, and 24 h after exposure. Using a two-way ANOVA, we found that most of the transcriptome changes were associated with time (Figure 7a, Supplementary Table S2). By comparison, there were about eight times fewer transcripts, of which the expression differed significantly between the sham and the exposed cells (Tables S3 and S4). QRT-PCR was used to confirm *Fos* expression changes from the time-dependent group, and *Zfp750* from the treatment-dependent group (Figure S4). Unsupervised hierarchical clustering performed simultaneously for the samples and differentially expressed genes revealed overall similarities between the sham- and nsPEF-exposed cells at any given time point, and the clustering of samples at 0 and 24 h. Sham- and nsPEF-exposed samples at 4 h formed a separate cluster (Figure 7b, upper panel). A relatively small group of 492 genes showed a clear overexpression in nsPEF-exposed cells compared to the sham (cluster 2; close-up in Figure 7b, lower panel). Gene ontology analysis revealed that the most positively enriched classes in this group were genes involved in the G-protein coupled receptor signalling pathway (fold enrichment 2.20, FDR = 0.009), transmembrane signalling receptor activity (fold enrichment 2.12, FDR = 0.003), and intrinsic components of the plasma membrane (fold enrichment 2.06, FDR = 0.027).

**Figure 8:**
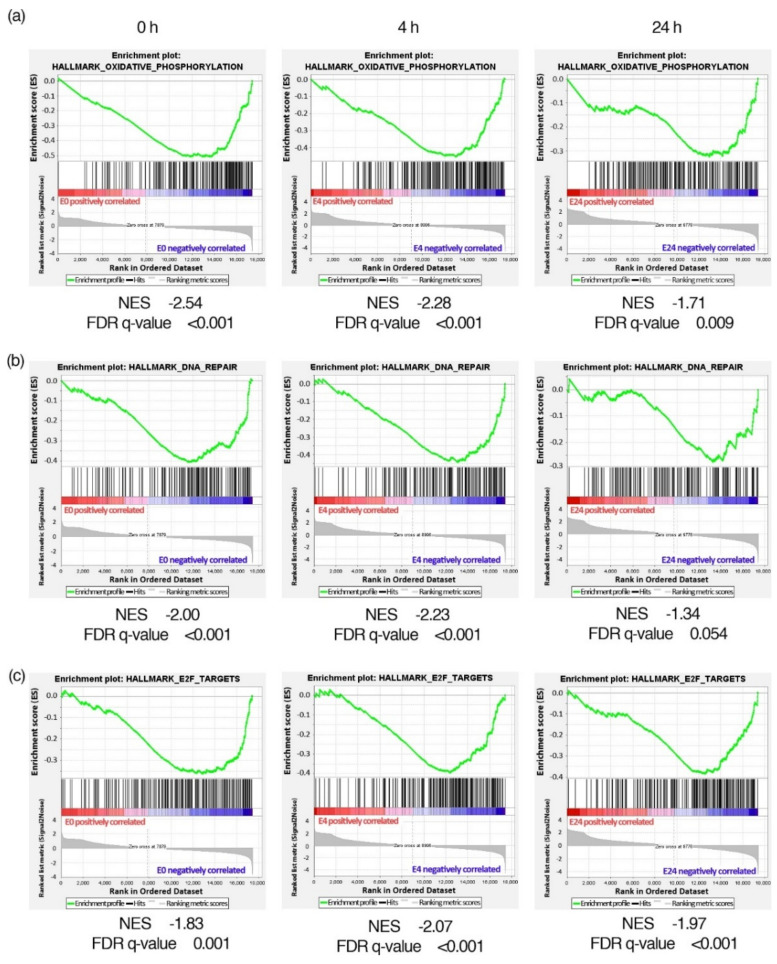
Gene set enrichment analysis for TM3 cells. E0: nsPEF-exposed cells, time 0 h; E4: nsPEF-exposed cells, time 4 h; E24: nsPEF-exposed cells, time 24 h. The most negatively enriched hallmark gene sets identified in the gene set enrichment analysis comparing nsPEF-exposed and sham samples at 0 h (left), 4 h (centre), and 24 h (right) time points. Normalised enrichment scores (NES) and false discovery rate (FDR) q-values are shown below the plots. (**a**) Hallmark: oxidative phosphorylation; genes encoding proteins involved in oxidative phosphorylation. (**b**) Hallmark: DNA repair; genes involved in DNA repair. (**c**) Hallmark: E2F targets; genes encoding cell-cycle-related targets of E2F transcription factors.

Alternative gene set enrichment analysis with the GSEA program revealed several positively and negatively enriched gene sets in cells exposed to nsPEF compared to the sham samples (Figure 8, Tables S5–S13). Gene sets positively enriched in the exposed cells were those coding for membrane-associated proteins, e.g., G protein-coupled receptors and voltage- or ligand-gated ion channels (Tables S5–S7), and genes downregulated by Kras activation (Tables S8–S10). The negative enrichment of genes involved in oxidative phosphorylation and DNA repair was most pronounced in cells exposed to nsPEF immediately and 4 h after exposure (Figure 8a,b; Tables S11 and S12). Negative enrichment in nsPEF-treated cells, especially at the four-hour time point, was also observed for gene sets involved in cell cycle and proliferation, e.g., E2F transcription factor targets (Figure 8c), genes involved in the G2/M checkpoint, and the genes essential for mitotic spindle assembly (Tables S11–S13).

Overall, transcriptome analysis revealed that nsPEF treatment induced fewer differences in gene expression than the changing conditions of a standard 2D cell culture over 24 h. After nsPEF treatment, overexpression was observed mostly for the genes whose products are plasma membrane proteins or the proteins closely located to the plasma membrane. Gene set enrichment analysis, which considers even small, but coordinated, changes in transcript levels from a functionally relevant group of genes revealed that nsPEF might have a negative, albeit probably transitory, impact on the expression of genes involved in oxidative phosphorylation, DNA repair, and cell proliferation.

## 3. Discussion

Our results show that disturbances in cell homeostasis due to nsPEF exposure can be transient, providing the cell is able to repair the damages quickly. On the basis of our observations, we hypothesize that a cell with a spare plasma membrane reserve can tackle the treatment with 60 nanosecond pulses at a 14 kV/cm electrical field without permanent perturbations in its cellular and molecular processes. In our experiments, it appeared that high-energy nanosecond pulses affected the plasma and mitochondrial membrane, diminished adhesion, induced breaks in DNA strands, disturbed the cell cycle, and altered the expression of some genes in the TM3 Leydig cells. However, after 24 h of exposure, the rate of TM3 cell proliferation, measured as both the metabolic activity and DNA content, remained at the same level as in the sham samples. Moreover, most of the adverse effects of nsPEF exposure concerned only a certain fraction of cells. The remainder of the cell population exhibited sham control characteristics.

It should be noted here that all the tests for every biological replicate (except for an adhesion test) were carried out in parallel on the same suspension of TM3 cells. In our opinion, this methodical approach can eliminate many factors that may disturb the test results, such as cell heterogeneity resulting from their successive passages to individual experiments, the errors in the cell densities in subsequent trials, and the inherent uniqueness of the predisposition of cells to a given stimulus resulting from their enormous intrinsic heterogeneity. Moreover, although TM3 cells grow as adherent monolayers, they were treated with nsPEF as suspensions to compare the results with numerous available publications.

The most prominent and easily noticed feature of altered cell physiology due to nsPEF is cell swelling. This phenomenon is caused by osmotic imbalance [24] due to the creation of nanopores [25] and the temporary permeabilization of the plasma membrane after monopolar nsPEF [26]. This leads to the leakage of ions from the cytoplasm to the external environment and vice versa. The nanopores may be transient and sealed by the cell quite fast or, in contrast, may be permanent, and the injured cell proceeds to death. The reversibility of the process, and the sealing of the pores in the cell membrane, are likely to occur only for pores of a diameter smaller than ~20 nm [27]. TM3 cells, in our experiments, enlarged their volume after treatment with nsPEF. Both increased cell diameters under an inverted microscope, and the higher FSC values in the flow cytometry clearly indicate the phenomenon of cell swelling.

In the search for the putative mechanism underlying this phenomenon, we have noticed, under SEM, an apparent change in the morphology of the cells treated with nsPEF. A fraction of enlarged cells lost microvilli from half, or the entire, cell surface. The microvilli of TM3 cells are hair-like projections of the plasma membrane stabilized by cross-linked actin fibres [28,29]. As previously shown, cell swelling may reduce their numbers [15] and modify the cell surface properties since microvilli serve as a membrane reservoir [30]. Under the influence of nsPEF, this phenomenon has been previously observed, but a stronger electric field and more pulses were used for the two human hepatocellular carcinoma (HCC) cell line treatments, and it finally led to additional thermal effects [31]. 

Caveolae [32], the plasma membrane invaginations, have a similar function, and both structures, upon physiologically relevant osmotic stress, unfold and compensate the plasma membrane tension [30]. However, considering the surface area occupied by both structures, and the large change in cell volume, the microvilli could have had a greater impact on maintaining the continuity of the membrane during swelling. The contribution of caveolae in the compensation of increased cell volume has been recently suggested [33]. The presence of caveolae was observed in the TEM images of the sham samples, in contrast to nsPEF-exposed cells, in our study, and we could not exclude such compensation mechanisms of the plasma membrane tension increase [34]. The presence of CAV1, a major protein of caveolae, has been demonstrated in TM3 Leydig cells [35]. Caveolae are not detectable in immune cells [32]. However, such cells, e.g., leukemic T-cell lymphoblasts (Jurkat cells), demonstrate intense cell swelling following nsPEF [36]. Instead, Jurkat cells have abundant microvilli [37], but the mechanism of microvilli sacrificing for their recovery after the plasma membrane permeabilization has not yet been studied. Caveolin1α participates in microvilli length regulation in Madin–Darby canine kidney cells [38] and, though not yet proven, such a mechanism can be hypothesized to participate in microvilli loss in TM3 Leydig cells. 

What is more, the phenomenon of cell swelling concerned only a fraction of TM3 cells in our study. The cytometric analysis of the cell volume expressed by FSC showed that only cells that do not uptake propidium iodide (the fraction devoid of large pores in the plasma membrane, i.e., pores bigger than 1.5 nm [39]) swelled following nsPEF. The portion of cells that died and acquired the pores with a diameter big enough for propidium iodide penetration did not increase in volume. Similar observations have already been reported [39]. Over time, the value of FSC decreased for cells with intact membranes, indicating the active mechanisms of pore sealing.

Apart from swelling, cell blebbing was also noticed in TM3 cells after nsPEF treatment in this study. The exact mechanism for the blebbing of cells exposed to the electric field remains unknown [40]. Although this phenomenon may be related to apoptosis, it does not always lead to cell death. According to one of the hypotheses, the blebbing could be the cell’s response to plasma membrane damage that cannot be spontaneously repaired by the cell [41,42]. The influx of positive ions through the damaged membrane (mainly calcium and sodium) causes the bending of the cell membrane and promotes the formation of bubbles [43]. Damaged membrane fragments are sequestered and ejected outside the cell in the form of extracellular vesicles (ectocytosis) [42]. It could occur upon the direct physical impact of impulses and the cellular response to water uptake by the Donnan-type colloid-osmotic mechanism [44]. This feature could make cells capable of regenerating after the plasma membrane permeabilization induced by nsPEF. 

Microarray analysis of TM3 cells confirmed that the genes encoding the membrane proteins were mostly differentially expressed between the sham and exposed samples at 240 min post nsPEF exposure (cluster 2.2, Figure 7b). Among 128 upregulated genes in the nsPEF-treated TM3 cells, eight coded for olfactory receptors, representatives of a large family of olfactory-specific G-protein coupled receptors [45]. Surprisingly, other upregulated genes, such as those encoding for gamma subunit 5 of the voltage-dependent calcium channel [46], contactin 4 [47], piezo-type mechanosensitive ion channel component 2 (*Piezo2*) [48], and neurexin I [49] are also mainly recognized as highly abundant in neural tissues. According to the RefEx dataset [50], these proteins are also expressed in mouse testis, although their functions remain elusive.

Microvilli could also mediate the adhesion, e.g., the attachment of epithelial cells to the endothelium [28,51]. We hypothesized that the reduction in microvilli density on the TM3 plasma membrane surface may diminish the adhesion of nsPEF-exposed cells. To the best of our knowledge, the present study is the first to describe diminished cell attachment following nsPEF exposure. Cell adhesion in vitro includes three phases: initial attachment, which is regulated by electrostatic interactions; flattening, which requires integrins binding to the extracellular matrix components; and finally, cell spreading, driven by actin cytoskeleton reorganization and the formation of focal adhesions [52]. In our study, most of the nsPEF-exposed cells remained round and loosely attached to the surface, as opposed to the sham cells showing signs of flattening 60 min after seeding, as evidenced by epifluorescence microscopy images. Thus, nsPEF treatment could have affected, or delayed, the first two phases of cell adhesion. This might be due to the impaired integrin function and/or extracellular matrix production in nsPEF-treated TM3 cells. Another explanation could be a change in the electrostatic properties of the cells due to the pronounced disturbance of the plasma membrane properties. The initial attachment of spherical TM3 cells with microvilli on their surface can be governed by the unspecific binding to polystyrene [53]. The presence of microvilli imposes specific electrostatic features on the cell surface [54] and could drive the initial adhesion phase.

While testing cell adhesion, we noticed that when calcein ester was added to the cells before nsPEF treatment, calcein fluorescence intensity decreased in most of the exposed cells in comparison to the sham. This was factored into the calculations of the percentage of cells adherent to surfaces: a fluorescence signal after washes was always normalized to the signal before washes in the same well. The efflux of negatively charged membrane-impermeable calcein molecules from preloaded cells after 6 ns electric pulses was described previously [55] and indicates the presence of pores in the plasma membrane. In our study, cells with a stronger calcein signal, presumably with or without a low number of pores, attached to the surface in a higher number. Together, these observations suggest that nanoelectroporation, and its impact on cell physiology, might be linked with impaired cell adhesion.

The diminished TM3 adhesion can also result from disturbances of the actin cytoskeleton after nsPEF exposure [56]. Other authors, who have studied the response of adherent cells to nsPEF, reported cell rounding and swelling, followed by actin cytoskeleton disassembly [57,58]. Although cell swelling was observed in our study, the effect of nsPEF on the actin structure of TM3 cells could have only been temporary and is reversible since visualization of actin fibres with a fluorophore-tagged phalloidin did not reveal any abnormalities in the nsPEF-treated cells at 10 min and 240 min postexposure. However, one should bear in mind that the actin-staining procedure required several washing steps that could have resulted in the removal of the most loosely attached cells devoid of microvilli, especially 10 min after treatment. More work is needed to fully understand the mechanism of decreased TM3 cell attachment after nsPEF exposure.

Cells may undergo apoptotic-, necrotic-, or autophagy-dependent programmed cell death under cellular stress conditions. In this work, the first two mechanisms were considered, and the presence of phosphatidylserine on the outer leaflet of the plasma membrane was monitored. Similar to the results of others [59,60], a significantly increased externalization of phosphatidylserine immediately after nsPEF treatment was demonstrated, when compared to the samples stained with fluorophores after nsPEF exposure and measured at 30 min. The first phenomenon was likely caused by membrane permeabilization, especially since the influx of Ca^2+^ from the external environment, as well as the release of calcium ions from the intracellular stores caused by nsPEF [7], make linking PS with AnV easier [61]. Some researchers also believe that negatively charged PS can be pushed from the inner to the outer leaflet of the membrane driven by an electric potential across the membrane [31]. Within 60 min, the proportion of viable AnV^-^/Pi^-^ cells did not decrease visibly; however, propidium iodide penetrated the AnV^+^ cells over time, increasing the proportion of the AnV^+^/PI^+^ cell fraction. This may indicate that cell death might occur by necrosis or secondary apoptosis as a delayed consequence of nsPEF exposure. Pakhomova et al. [62] underline the important role of necrosis in cell responses to a pulsed electric field. After nsPEF exposure, and calcein AM and ethidium homodimer-1 staining, some cells with compromised membranes following nsPEF exposure were also visible under CLSM. The number of damaged cells decreased after 240 min of incubation in standard conditions when a fresh medium with serum was added to the culture. It might indicate that the membranes were repaired enough to prevent ethidium homodimer-1 penetration to the cell interior.

The membrane permeabilization resulting from the nsPEF treatment could also affect mitochondria. The mitochondrial membrane potential, the gradient of the electric potential on the inner mitochondrial membrane (ΔΨm), is a crucial element for the synthesis of ATP through oxidative phosphorylation. Damage to the mitochondrial membrane leads to disturbances in the energy metabolism process. It has already been pointed out that submicrosecond electroporation decreases mitochondrial membrane potential [63,64], and this disturbance stems from the activation of the calcium-dependent pathways rather than direct mitochondrial membrane permeabilization [65]. The mitochondrial membrane potential dropped after the nsPEF treatment of TM3 cells in this study; however, this phenomenon concerned only a fraction of the nsPEF-exposed cells. This confirms a well-known phenomenon of mitochondrial heterogeneity [66]. Since the breakdown of mitochondrial membrane potential within the cell population may indicate the damage extent [67], we concluded that nsPEF treatment did not lead to serious cell injury within the overall exposed cell population. This observation also points to a phenomenon of the heterogeneous cell response to nsPEF. The undisturbed proliferation of cells in the next 24 h of incubation might confirm a minor extent of damage in these experiments. Similar undisturbed proliferation was also shown for porcine bone marrow mesenchymal stem cells after nsPEF treatment [68].

The microarray-derived results of this study show a lower expression of the genes involved in oxidative phosphorylation after the nsPEF stimulation of TM3, especially immediately after, and 4 h, post-treatment. The functional mitochondrial electron transport chain (ETC) is responsible for creating an electrochemical proton gradient across the inner mitochondrial membrane and for reactive oxygen species (ROS) generation. Diminished ETC activity, due to the reduced production of its constituents, could lead to decreased mitochondrial transmembrane potential and lower levels of superoxide production in mitochondria, as was observed in our study. 

A few previous studies demonstrated that nsPEF might affect DNA and nuclear processes within mammalian cells, as summarized by Schoenbach [1]. Therefore, the generation of breaks in DNA strands upon nsPEF treatment was monitored in our experiments. The highest number of comets was observed at stage B, indicating that the damage was not severe. However, the pattern of the distribution of the comet stages in the exposed cells and the cells treated with etoposide (the positive control) was similar. On the basis of the above observation, it can be assumed that the mechanism of DNA damage might be similar. Etoposide is a topoisomerase II inhibitor that induces double-strand breaks (DSBs) in the cells [69]. However, Qian et al. [70] demonstrated that both single-strand breaks (SSBs) and DSBs, after nsPEF exposure, might occur in the cells. The SSBs and DSBs stimulated by nsPEF were detected in the comet assay performed in both neutral and alkaline conditions [69], and significant DNA damage was noticed just 90 s after the nsPEF stimulation [71].

The cell usually repairs its damaged DNA if changes are not severe. Following this, damaged DNA could be repaired over an hour after the nsPEF stimulation of murine melanoma cells [71]. Similar results were obtained for Jurkat cells [72]. In our study, the neutral comet assay was carried out within half an hour after the nsPEF stimulation; therefore, we assumed that DNA repair processes had hardly started. We also demonstrated a relatively low level of DNA damage in the exposed group compared to the positive control. The DNA damages induced in TM3 cells following the nsPEF exposure were probably repaired within a few hours, which was analogous to the results obtained by the other authors cited above. It was also possible because of a lack of cells in comet stage E, i.e., with total nuclear DNA damage.

In previous publications, the influence of nsPEF on the cell cycle has often been ignored, although it affects the cell’s sensitivity to the injury, e.g., DNA damage, or inhibits activation of the repair processes. The exposure of cells of the same origin, but at different cycle phases, can produce divergent results. Jurkat cells appeared to be more sensitive to nsPEFs in the G2/M phase than in the G1/S phase [73]. Monitoring cell distribution at a specified cell cycle phase, we did not observe cell cycle arrest in the G2/M phase, unlike previous studies performed on Chinese hamster ovary (CHO) cells [73,74]. Instead, a lower proportion of the G2/M phase was visible at 4 h after treatment compared to the sham samples. We also noted a markedly increased percentage of cells in the subG1, and a decrease in the S phase in nsPEF-treated cells compared to the sham samples. These changes might indicate the ongoing process of apoptosis in a certain population of cells. After 24 h, a fraction of cells entering the DNA synthesis phase and cell division returned to the sham levels. This was accompanied by a decrease in the number of cells in the G0/G1 phase. In the G1 phase, the cells grow, multiply organelles, and synthesize proteins while preparing to divide. Cells in the G1 phase are characterized by relatively high membrane fluidity, and associated with a high synthesis and degradation of phospholipids [75]. This property may reduce the sensitivity of cells to nsPEF and regenerate cell membranes more easily. This phenomenon might have influenced the susceptibility of TM3 cells with a large excess of the plasma membrane to the pulsed electric field.

The observed changes in cell cycle phases were reflected in the overall expression of gene sets involved in cell cycle progression, which were negatively enriched in nsPEF-treated cells as genes involved in the G2/M checkpoint and those essential for mitotic spindle assembly. Among the negatively enriched genes, there were also E2F transcription factor targets involved in DNA replication and repair, cell cycle regulation, and checkpoints [76]. Again, the most notable changes were observed 4 h after nsPEF treatment.

Transcriptome analysis, in our study, had the unique property of including the immediate, early (4 h), and late response (24 h) of cells to nsPEF exposure, in contrast to previously published studies analysing the differential gene expression of the immediate (at 0.5 and 1 h) [77] and early (at 4 h) cell responses [78,79]. This allowed us to trace the nsPEF-related variations in gene expression for the first time over a more extended period. Both types of performed analyses, i.e., differential gene expression and gene set enrichment, showed that most of the nsPEF-induced changes in gene expression were transient in our experiments. This was evidenced by the fact that, within a cluster of genes overexpressed due to nsPEF treatment (cluster 2 in Figure 7b), we could distinguish separate subclusters that showed a peak of gene expression at 0, 4, or 24 h. Similarly, we observed fluctuations in gene set expression over time. For example, the negative enrichment of genes involved in DNA repair was most prominent at 4 h after nsPEF exposure, whereas after 24 h, the overall expression levels of genes in this set in nsPEF-exposed cells were more comparable to the sham samples. The genes in nsPEF-exposed Jurkat cells were also transiently overexpressed; the differential expression of the genes involved in biochemical pathways was most prominent 30 min after treatment, whereas, at 60 min, the cell cycle and gene transcription pathways were the most affected processes after nsPEF treatment [77]. On a smaller scale, an initial overexpression due to nsPEF exposure, followed by a drop in protein levels up to 12 h after exposure, were also observed for proteins involved in the MAPK signalling pathway in Jurkat and U937 cells [79]. Our results emphasize the importance of studying gene expression after nsPEF treatment over a longer period of time in order to better understand the dynamic responses to the nanosecond pulsed electric field.

Interestingly, the ANOVA and hierarchical clustering analyses revealed that, in our model, transcriptome variations due to nsPEF were much more subtle than the changes that occurred in cells during a standard culture. Samples clustered preferentially over time, not treatment, with the cells harvested at 4 h forming a separate cluster, showed the most distinctive expression profile among the sample types studied. The group of genes overexpressed at 4 h (cluster 4, Figure 7b) showed the enrichment of proteins involved in ribosome biogenesis and translation initiation. The genes with lower expression at 4 h (cluster 1, Figure 7b) encoded for proteins participating in DNA replication, DNA repair, and cell cycle progression. This represents an expected sequence of events when healthy cells grow and proliferate: ribosome assembly and protein production precede progression through the cell cycle and division [80]. Other noteworthy time-related changes included a high overexpression of *Fos* (FBJ osteosarcoma oncogene), *Jun* (jun proto-oncogene), and *Egr1* (early growth response 1) immediately after nsPEF, both in the sham- and nsPEF-exposed cells. This finding was surprising because previous works strongly associated increased expression of these genes with nsPEF treatment in Jurkat cells and fibroblasts [77,78,79,81]. *Fos*, *Jun,* and *Egr1* are well-characterized immediate-early response genes whose transcription is activated within minutes after stimulation with extracellular or intracellular cues (e.g., cytokines, growth factors, shear stress, or UV damage) [82,83,84]. Their expression is regulated by several signalling pathways, including the RHOA/ROCK, ERK MAPK, P38 MAPK, or PI3K pathways [82]. The increased expression of *Fos*, *Jun*, and *Egr1* at 0 h in our experiments could reflect a response to the stress imposed by prolonged sample preparation (cell detachment, centrifugation, filtering, and exchange of medium) prior to nsPEF treatment. After 4 h and 24 h, when cells were harvested quickly, no effect on the expression of these genes was observed. NsPEF did not seem to elicit reaction involving immediate-early response genes in our model, presumably contrary to other types of cells [78,79].

Despite the abovementioned discrepancies with previous publications, some common observations regarding gene expression changes due to nsPEF exposure were noticed in our experiments. Overexpression of genes coding for membrane-associated proteins in nsPEF-exposed cells, even if different for individual proteins, was consistent with other studies [28,79]. The downregulation of genes involved in the cell cycle and DNA repair was also previously identified in Jurkat cells following nsPEF treatment [77]. Nevertheless, the magnitude of changes seemed to be higher in the immune cells since significant differential expression could be detected for individual genes. In our experimental model, the coordinated, but subtle, changes were detectable only at the level of gene sets. The observed differences might stem from the higher sensitivity of Jurkat cells to nsPEF exposure [79] compared to TM3 cells exhibiting the capability to repair nsPEF-induced damage.

## 4. Materials and Methods

### 4.1. Cell Culture

Leydig TM3 mouse testicular cells (ATCC CRL-1714, Manassas, Virginia, USA) were propagated in a composed medium, DMEM/F12 (Dulbecco’s modified Eagle’s and Ham’s F12 medium, 1:1, Lonza, Basel, Switzerland), with supplements (Lonza, Basel, Switzerland): 5% horse serum, 2.5% fetal bovine serum, and antibiotic/antimycotic in 5% CO_2_ at 37 °C until semiconfluent. The cells were detached with ReagentPack^TM^ Subculture Reagents (Lonza, Basel, Switzerland). For the experiments, TM3 cells, at the density of 1.5 × 10^6^ ± 2.0 × 10^5^ cells/mL, were washed twice with PBS (Gibco^®^, Waltham, MA, USA), resuspended in 1 × Annexin V binding buffer (BB, BD), passed through a 40-µm nylon cell strainer filter (Corning, NY, USA), and aliquoted into electroporation cuvettes (0.4 cm gap size width, Invitrogen™, Waltham, MA, USA) in a volume of 200 µL per sample. The density of the cell suspension in cuvettes was 1 × 10^6^ cells/m. Exposure to nsPEF was carried out at room temperature. The cells remained in the cuvette only for the duration of the exposure and were tested immediately afterwards.

### 4.2. Exposure to nsPEF

A compact 4-kV pulse generator that combined two types of switches, insulated-gate bipolar transistors (IGBTs), and a spark gap based on Blumlein-line architecture, were used in the experiments. This approach reduced the size and cost of the generator and increased its performance and the controllability of the generated pulses. A full description of the architecture and operation of the pulse generator has been described previously [85]. The generated pulses had a quasi-rectangular shape, a 60 ns pulse width, and 5.5 kV amplitude, which corresponded to an electric field within the cuvette of 14 kV/cm. The repetition rate was 0.44 pulses per second, and the duration of the whole treatment was 180 s. The shape of the pulse, its repeatability, and other exposure parameters are presented in Figure S1a–c. The volume of the medium with cells treated with nsPEF was calculated precisely to get a matched terminal impedance for the pulse generator. Current and voltage waveforms were measured under exposure conditions with a WaveRunner 640 Zi oscilloscope (Teledyne Lecroy, Chestnut Ridge, NY, USA). The probes used for the voltage and current measurements were P6015A and P6021 (Tektronix, Beaverton, Oregon, USA), respectively. The sham and nsPEF treatments were performed at room temperature. The buffer’s temperature during the electroporation was monitored with a fibre optic probe (PRB-400, OSENSA, Burnaby, BC, Canada). The probe was placed between the electrodes. The health of the TM3 cells was then assayed simultaneously by several techniques, listed below in each experiment.

### 4.3. Cell Diameter

The sham and nsPEF-exposed cells were seeded into 24-well plates (1 mL, 10^5^ cells/mL) immediately, or 240 min, after treatment, and the cell images were recorded using an inverted optical microscope (Primo Vert, Carl Zeiss, Oberkochen, Germany). Cell diameters were measured using ZEISS ZEN software (Carl Zeiss Ltd., Oberkochen, Germany). Avoiding the error caused by optical phenomena, only cells located in the microscope’s focal plane with a visible border of the cell membrane were counted. Moreover, the relative cell size was monitored with the BD (NJ, USA) FACSAria™ III flow cytometer (BD FACSDiva software version 10.0, NJ, USA, with a minimum of 10,000 cells per sample) at 0, 30, 60, and 240 min after nsPEF exposure and recorded as a fold change of the forward scatter (FSC) parameter values relative to the sham.

### 4.4. Electron Microscopy

TM3 cells were fixed by paraformaldehyde (PFA, Sigma-Aldrich, Burlington, MA, USA) with a glutaraldehyde (GA, Sigma-Aldrich, Burlington, MA, USA) mixture (SEM: 4% PFA with 0.4% GA; TEM: 2% PFA with 2.5% GA). Then, cells were rinsed with 0.1 M cacodylate buffer (Sigma-Aldrich, Burlington, MA, USA) and postfixed with 1% osmium tetroxide (Sigma-Aldrich, Burlington, MA, USA). The dehydration of the samples was performed in increasing ethanol concentrations (30–99%). The cells for the scanning electron microscopy (SEM) observation were incubated with increasing concentrations of acetone (30–100%), filtered through the polymer membrane, and dried in a critical point dryer (Leica, Wetzlar, Germany, EM CPD300). Next, the samples were platinum-coated using a sputter coater (Leica, Wetzlar, Germany, EM ACE200) and observed in high vacuum mode with the Everhart-Thornley Detector (ETD) on a scanning electron microscope, STEM Quanta FEG250 (FEI, Oregon, USA). For transmission electron microscopy (TEM), the cells were rinsed in propylene oxide (Acros Organic, Waltham, MA, USA) after ethanol incubation and stained with 1% uranyl acetate (Ted Pella, Redding, CA, USA) in 70% ethanol. Next, the cells were embedded in Epon resin (Sigma-Aldrich, Burlington, MA, USA) with propylene oxide and then in pure Epon resin. After polymerization (2 days in 60 °C), the samples were cut into thin sections using an ultramicrotome, and TEM images were obtained with the transmission electron microscope JEM 1400 (JEOL Co., Tokyo, Japan) at 80 kV.

### 4.5. Adhesion

The adhesion of TM3 cells was assayed by a wash assay after their staining with 4 µM Calcein AM (Thermo Fisher Scientific, Waltham, MA, USA) in DMEM/F12 medium without the sera (DMEM/F12-). After nsPEF treatment, the stained cells were centrifuged at room temperature for 5 min at 150× *g*, resuspended in DMEM/F12-, seeded into 96-well plates (100 µL, 5 × 10^4^ cells/well), and incubated at 37 °C, 5% CO_2_. After 40 and 60 min, the cells were washed twice with warm DMEM/F12- medium; during washes, the plates were vortexed for 1 min at 500× *g* on a IKA^®^ (Burlington, MA, USA) MS 3 digital shaker. Before and after washes, cell images were recorded using the Nikon Eclipse Ts2R inverted microscope with epifluorescence. Fluorescence intensity was measured with a CLARIOstar reader (BMG Labtech, Ortenberg, Germany) with the following settings: excitation, 483/14 nm; dichroic filter, 502.5 nm; emission, 530/30 nm; measurement mode, top reading; focal height, 4.7 mm; scan mode, orbital averaging; scan diameter, 4 mm; number of flashes per well, 20. The unstained cells were used as a background control. The fraction of adherent cells was calculated by dividing the blank subtracted fluorescence intensity values before and after washes. 

### 4.6. Filamentous Actin

The cells were put into 4-well chamber slides (Zell-Kontakt GmbH, Nörten-Hardenberg, Germany) covered by polylysine (10^4^ cells/well) immediately after treatment. After 10 or 240 min at 37 °C, the samples were fixed with formaldehyde (4%, 15 min), followed by 0.1% Triton X-100 in PBS (5 min), with washes in between. Then, cells were incubated with 1% BSA (20 min), stained with Alexa Fluor™ 488 Phalloidin (Invitrogen™, Waltham, MA, USA), counterstained with Hoechst (Invitrogen™, Waltham, MA, USA) according to manufacturer’s protocols, and visualized under a LSM 700 Axio Observer Z1 Zeiss confocal laser scanning microscopy (CLSM, Carl Zeiss, Oberkochen, Germany).

### 4.7. Phosphatidylserine Externalization

Alexa Fluor 488-conjugated annexin V (5 μL) and propidium iodide (PI) (5 μL) were added to 100 μL of TM3 cells in 1 × BB, at the density of 1–2 × 10^6^ cells/mL, either just before or immediately after nsPEF exposure. Sham-exposed cells were used as negative controls. The samples were analysed by the BD (NJ, USA) FACSAria™ III flow cytometer using the BD FACSDiva software version 10.0 (NJ, USA), with a minimum of 10^4^ cells per sample. The cell populations were distinguished as: living (annexin V-negative, PI-negative); apoptotic (annexin V-positive, PI-negative); late apoptotic/necrotic (annexin V-positive, PI-positive); and dead (annexin V-negative, PI-positive). The cells were gated using TM3 cells, either untreated or treated with formalin or camptothecin (350 μM); the gates were set in the green 530/30 channel for Alexa Fluor 488, and in the yellow 585/45 channel for PI. The excitation was performed with an argon laser (488 nm).

### 4.8. Mitochondrial Membrane Potential

The Mitochondria Staining Kit (Sigma-Aldrich, Burlington, MA, USA) was performed according to the manufacturer’s protocol. The cyclic polymer valinomycin, and carbonyl cyanide m-chlorophenyl hydrazine (CCCP), were used as positive controls. After excitation at 488 nm, the fluorescence emission intensity in the green 530/30 and yellow 585/15 channels were recorded with the BD (NJ, USA) FACSAria™ III using BD FACSDiva software 10.0 (NJ, USA). The ratio of yellow to green fluorescence was calculated.

### 4.9. ROS Formation

CellROX^®^ Orange Reagent, and MitoSOX™ Red Mitochondrial Superoxide Indicator (Invitrogen™, Waltham, MA, USA), at concentrations of 5 μM, were used to stain 10^4^ cells in 96-well plates and 8-well chambered coverglass. The cells were counterstained with Hoechst (Invitrogen™, Waltham, MA, USA). The stainings were performed according to manufacturer protocols. After washes with warm PBS, the fluorescence intensity was measured with a BMG Labtech (Ortenberg, Germany) CLARIOstar reader, with an excitation/emission of 545/565 nm for CellROX^®^ Orange Reagent, and 510/580 nm for MitoSOX™ Red. The cells on the coverglass were visualized under CLSM.

### 4.10. Comet Assay

The OxiSelect™ Comet Assay Kit (Cell Biolabs, San Diego, USA) was used. TM3 cells, at a density of 1 × 10^5^/mL, were washed and suspended in 10% OxiSelect™ Comet Agarose. Then, 75 μL of the suspension was pipetted into each well of the OxiSelect™ 3-Well Comet Slides. According to the manufacturer’s instructions, the slides were processed and finally electrophoresed at 1.3 V/cm for 20 min. The comets stained with ethidium bromide (20 µg/mL) were imaged using a Nikon (Tokio, Japan) Eclipse Ci-L epifluorescence microscope, and at least 70 cells per slide for each group were recorded using the Nikon DSFi1-U2 camera equipped with the Nikon NISF software. The etoposide-treated cells (350 µM, Sigma-Aldrich, Burlington, MA, USA) were used as a positive control. The DNA damage was analysed using the CaspLab^®^ software (2020, http://casplab.com, (accessed on 15 August 2021)). The percentage of DNA in the comet tail was quantified, and the tail moment was calculated as the percentage of DNA in the tail multiplied by the length of the tail. Additionally, the nuclei of the TM3 cells were classified into five categories based on the quantity of DNA in the tail [86].

### 4.11. Cell Proliferation

Cells were seeded in 96-wells plates (100 µL, 2 × 10^4^/mL) and, after 24 h of incubation in complete medium under culture conditions, the metabolic activity and DNA content of the TM3 cells were measured with PrestoBlue Cell Viability Reagent (Invitrogen™, Waltham, MA, USA) and CyQuant NF Cell Proliferation Assay (Invitrogen™, Waltham, MA, USA), respectively. The tests were performed according to the manufacturer’s protocols. Fluorescence intensity was measured with a BMG Labtech (Ortenberg, Germany) CLARIOstar reader (excitation/emission: 557/593 nm for PrestoBlue, and 485/530 nm for CyQuant assay).

### 4.12. Cell Cycle

The sham- and nsPEF-treated cells were seeded into 24-well plates with complete medium, and incubated at 37 °C, 5% CO_2_ for 4 and 24 h. After harvesting, 10^5^ cells were used to control the cell cycle progression using the Tali^®^ Cell Cycle Kit (Thermo Fisher Scientific, Waltham, MA, USA) and the Tali Image-Based Cytometer (Invitrogen™, Waltham, MA, USA). The kit was used according to the manufacturer’s protocol. The fraction of cells in each phase of the cell cycle was determined using the Tali Image-Based Cytometer Firmware ver. 2.2 software (Invitrogen™, Waltham, MA, USA).

### 4.13. Viability of Cells

Sham- and nsPEF-exposed cells were aliquoted into 24-well plates with complete medium (1 mL, 1 × 10^5^ cells/mL) immediately after treatment. The LIVE/DEAD™ Viability/Cytotoxicity Kit (Invitrogen™, Waltham, MA, USA) was used according to the manufacturer’s protocol. The cells were visualized under CLSM at 15 and 240 min after exposure. 

### 4.14. RNA Isolation

Total RNA was isolated from frozen cell pellets using the RNeasy Mini Kit (Qiagen, Hilden, Germany) (including the on-column DNase I treatment). RNA was quantified using the QuantiFluor RNA System (Promega, Madison, WI, USA) and the Quantus Fluorometer (Promega, Madison, WI, USA). RNA quality was assessed with 2100 Agilent Bioanalyzer and Agilent RNA 6000 Nano Reagent (Agilent Technologies, Santa Clara, CA, USA). The RIN (RNA integrity number) ranged from 8.9 to 10.0.

### 4.15. Microarrays

Cyanine-3 (Cy3), labelled cRNA, was prepared from 100 ng of total RNA using the Low Input Quick Amp Labelling Kit, One Color (Agilent Technologies, Santa Clara, CA, USA), followed by the RNeasy Mini Kit (Qiagen, Hilden, Germany) column purification. RNA Spike-In, One Color, was added to the total RNA as an internal control. cRNA quality and quantity were assessed using the 2100 Agilent Bioanalyzer and Agilent RNA 6000 Nano Reagent (Agilent Technologies, Santa Clara, CA, USA). An amount of 600 ng of Cy3-labeled cRNA was processed following the manufacturer’s protocol, hybridized to Sure Print G3 Mouse Gene Expression v2 8 × 60 K microarrays (G4852B, Agilent Technologies, Santa Clara, CA, USA), and scanned with a SureScan Microarray Scanner. Scanned images were analysed with the Feature Extraction Software 12.0, using default parameters (protocol: GE1_1200_Jun14, grid: 074809_D_F_20190110) to obtain the background-subtracted and detrended Processed Signal intensities. The final data have been deposited in the NCBI’s Gene Expression Omnibus [87] and are accessible through GEO Series accession number GSE155355 (https://www.ncbi.nlm.nih.gov/geo/query/acc.cgi?acc=GSE155355 (accessed on 2 June 2021)). Microarray data were preprocessed with GeneSpring 14.9 (Agilent Technologies, Santa Clara, CA, USA), normalized by a percentile shift (75th percentile), and the baseline was set to the median of all samples. Differential gene expression analysis for the genes detected in at least two out of four biological replicates in any condition was performed in the GeneSpring using a two-way ANOVA with interaction, and Benjamini–Hochberg FDR multiple testing correction. Genes with *p* < 0.05 were reported. Subsequently, unsupervised hierarchical clustering was performed for all ANOVA results and the biological replicates with the following settings: similarity measure, Euclidean; linkage rule, Ward’s. Gene Ontology statistical overrepresentation tests were performed for clusters of genes with a similar expression pattern using PANTHER v. 16 (2021, http://pantherdb.org/ (accessed on 27 May 2021)) [88]. Fisher’s exact test and a correction based on the false discovery rate calculation were applied. Preprocessed microarray data were also used for gene set enrichment analysis with GSEA software [89,90] and gene sets from the Molecular Signatures Database (2020, https://www.gsea-msigdb.org/gsea/msigdb/index.jsp (accessed on 27 May 2021)) [91]: H collection—hallmark gene sets [92]; and C5 collection—Gene Ontology gene sets. The settings were as follows: probes for the same gene were collapsed; the statistical significance was determined with gene set permutation (no. of permutations: 1000), and the gene set size was 15-500 for H, and 50-500 for the C5 collection. The false discovery rate (FDR) cut-off was set at 0.05.

### 4.16. Quantitative Reverse Transcription Polymerase Chain Reaction (QRT-PCR)

To remove all traces of genomic DNA before QRT-PCR, 1 µg of total RNA was treated with TURBO DNase (TURBO DNA-free kit, Thermo Fisher Scientific, Waltham, MA, USA) at 37 °C for 30 min. RNA concentration was measured with the QuantiFluor RNA System (Promega, Madison, WI, USA) and Quantus Fluorometer (Promega, Madison, WI, USA) after DNA removal. 10 ng of total RNA was used for one-step QRT-PCR with Brilliant III Ultra-Fast SYBR Green QRT-PCR Master Mix (Agilent Technologies, Santa Clara, CA, USA), and 300 nM of gene-specific forward and reverse primers. QRT-PCR was performed using the Agilent (Aligent Technologes, Santa Clara, CA, USA) Aria Mx instrument with the following steps: reverse transcription (10 min at 50 °C); hot start (3 min at 95 °C); amplification (5 s at 95 °C, and 10 s at 60 °C, 40 cycles); melt curve (30 s at 95 °C, 30 s at 60 °C, 30 s at 95 °C, resolution 0.5 °C, soak time 5 s). Each sample was run in duplicate. Primer pairs were selected among experimentally validated primers from PrimerBank (2018-2019, https://pga.mgh.harvard.edu/primerbank/ (accessed on 14.12.2020)) [93,94]; PrimerBank IDs were: 31981890a1 (*B2m*, NM_009735); 7106439a1 (*Tubb5*, NM_011655); 6753894a1 (*Fos*, NM_010234); and 30520225a1 (*Zfp750*, NM_178763). Primers were synthesized by the DNA Sequencing and Oligonucleotides Synthesis service (http://www.oligo.pl/ (accessed on15.12.2020)). Relative gene expression was calculated with the ΔΔCq method [95] and shown as log2 values. The geometric mean of *B2m* and *Tubb5* expression was used as a normalization factor [96].

### 4.17. Statistical Analysis

The Kolmogorov–Smirnov test was used to compare distributions between populations; the test results were calculated using the Quest Graph™ Kolmogorov–Smirnov (K-S) Test Calculator (2020, AAT Bioquest Inc., Sunnyvale, CA, USA, https://www.aatbio.com/tools/kolmogorov-smirnov-k-s-test-calculator (accessed on 12 January 2021)). The Student’s t-test and nonparametric Mann–Whitney U test were used to compare two sets of measurements; these data were analysed with the Statistica version 13 (TIBCO Software Inc., Palo Alto, CA, USA). Fitting the size of the cells to a Gaussian distribution was done with the same software. At least three replications were processed in each experiment. Details on the number of experiments, and the biological and technical replications, are provided under the figures presenting the results. A chi-square test was applied to compare the proportions of cells in a given cell cycle phase between sham- and nsPEF-exposed cells. Calculations were performed using a chi-square calculator, retrieved from https://www.socscistatistics.com/tests/chisquare2/default2.aspx (accessed on 12 January 2021) (2020). In all statistical tests, the significance level (α) was set to 0.05. The obtained p values are given in the text or in the Figure legends. The description of the microarray data statistical analyses is provided in 1.15. Microarrays section of Methods.

## 5. Conclusions

This study aimed to evaluate the utility of nanosecond pulsed electric field (nsPEF) for the electroporation of testosterone-producing Leydig cells for medical applications in reproductive biology. Two conditions should be met when applying this technique for the successful electrotransfer of therapeutic molecules into cells: transient permeabilization of the plasma membrane, and a minimal detrimental effect on cell viability and metabolism. 

The formation of nsPEF-induced pores was assessed in this study by monitoring the flow of the membrane-impermeable molecules through the cell membrane with many different techniques. Considering the results, we can conclude that the exposure of Leydig TM3 cells to nanosecond electric field pulses, with an intensity of 14 kV/cm and a duration of 60 ns, is an effective tool for forming pores in these cells. Moreover, our results point to a rapid sealing of the pores, indicating the transient effect of nsPEF on TM3 membrane permeability.

To detect possible cellular stressors after exposure to nsPEF, we included a range of analyses from observations of the morphology by assessing the physiological processes and DNA damage to transcriptome analysis in TM3 cells. Temperature measurements during the exposure allowed us to conclude that the thermal effect of nsPEF at the conditions used was negligible. Despite that, we observed cell swelling, the loss of microvilli from the cell surface, diminished adhesion, signs of apoptosis and necrosis, reduced mitochondrial membrane potential and superoxide production in mitochondria, DNA damage, and cell cycle disturbances immediately after applying the nanosecond pulsed electric field. It was accompanied by subtle changes in the gene expression, detected at the levels of functionally or spatially connected gene sets (e.g., oxidative phosphorylation gene set, proteins located in the cell membrane) rather than individual genes. However, these changes did not alter TM3 cells in a permanent way, as indicated by their viability at the later time points, which was comparable to the sham-treated control. Moreover, the response of TM3 cells to nsPEF exposure was heterogeneous. We observed most of the abovementioned changes only in a subpopulation of treated cells. This allows for presuming that no harmful unrepairable molecular events occur in the electroporated Leydig TM3 cells. We can also conclude that cellular processes after nsPEF treatment, at various time points, should be monitored in future studies, especially when the observed changes are short-lived or undergo rapid remission.

We also hypothesized that certain features of the morphology and physiology of the TM3 cells influenced their specific sensitivity to nsPEF. The critical features of TM3 cells, which remained viable after nsPEF treatment, could be microvilli on their surface and the cell population’s observable heterogeneity in response to the pulsed electric field. Future studies could test these assumptions on a more homogenous TM3 cell population (e.g., synchronized). The detection of specific proteins involved in membrane permeabilization and the repair processes, cell cycle progression, and DNA repair, after nsPEF exposure, should enrich further research. Such studies would undoubtedly clarify the observed phenomenon of repairing damage caused by nsPEF in TM3 cells.

In summary, our research focused on the effects of the nanosecond pulsed electric field alone, without the thermal component. This approach allowed us to test the potential applicability of the tested nsPEF parameters to transiently permeabilize the cell membrane with the least possible impact on cell homeostasis. The established exposure parameters of the TM3 Leydig cells can be used for further studies on reversible electroporation, and for the potential application of this method to targeted therapy, such as small RNA electrotransfer. In future research using specific small RNAs, one should verify whether the electroporation parameters used here ensure the sufficient effectiveness of the given therapy.

## Data Availability

The microarray data are deposited in NCBI’s Gene Expression Omnibus and are accessible through GEO Series accession number GSE155355 (https://www.ncbi.nlm.nih.gov/geo/query/acc.cgi?acc=GSE155355) (accessed on 2 June 2021).

## Supplementary Materials

The following are available online at https://www.mdpi.com/article/10.3390/ijms222011236/s1.
