# Supplementary material for: Nanosecond Pulsed Electric Field Only Transiently Affects the Cellular and Molecular Processes of Leydig Cells
Source: Int J Mol Sci. 2021 Oct 18;22(20):11236. doi: 10.3390/ijms222011236 (PMC8541366; doi:10.3390/ijms222011236)
Supplement: Supplementary file 1 [file ijms-22-11236-s001.zip › Kasprzycka at al. Supplementary_Figures.pdf]

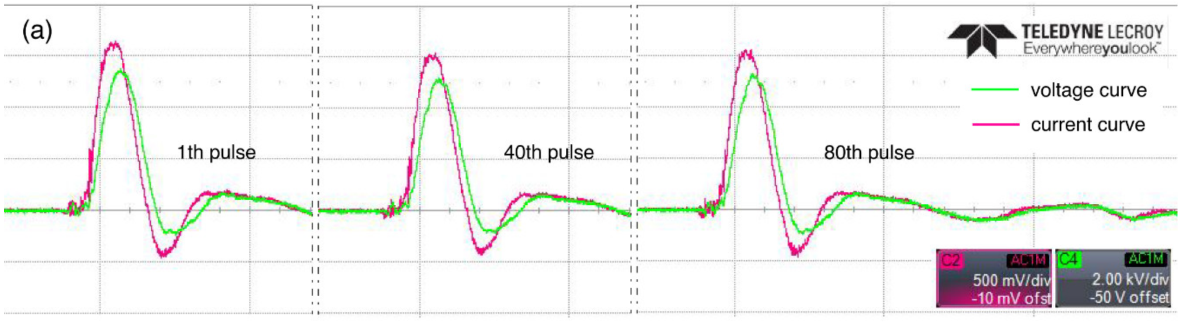

(b)

voltage curves			current curves
amplitude (kV)	rising time (ns)	width (ns)	amplitude (A)
5.5 ± 0.2	24.7 ± 1.4	59.6 ± 0.3	2.8 ± 0.2

(c)

field (kV/cm)	distance between electrodes (mm)	repetition rate (pps)	number of pulses	duration of exposure (s)
14	4	0.44	80	180

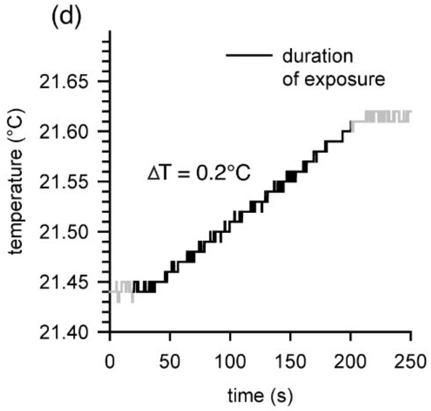

Figure S1. Parameters of the exposure to nsPEF. (**a**) Voltage and current waveforms simultaneously measured under exposure conditions. (**b**) Electric parameters of pulses measured under exposure conditions (±SD, n = 80 measurements) demonstrating no significant distortion in shapes and amplitudes. (**c**) Parameters of exposure. (**d**) Temperature effect control. No significant change in temperature throughout the exposure cycle.

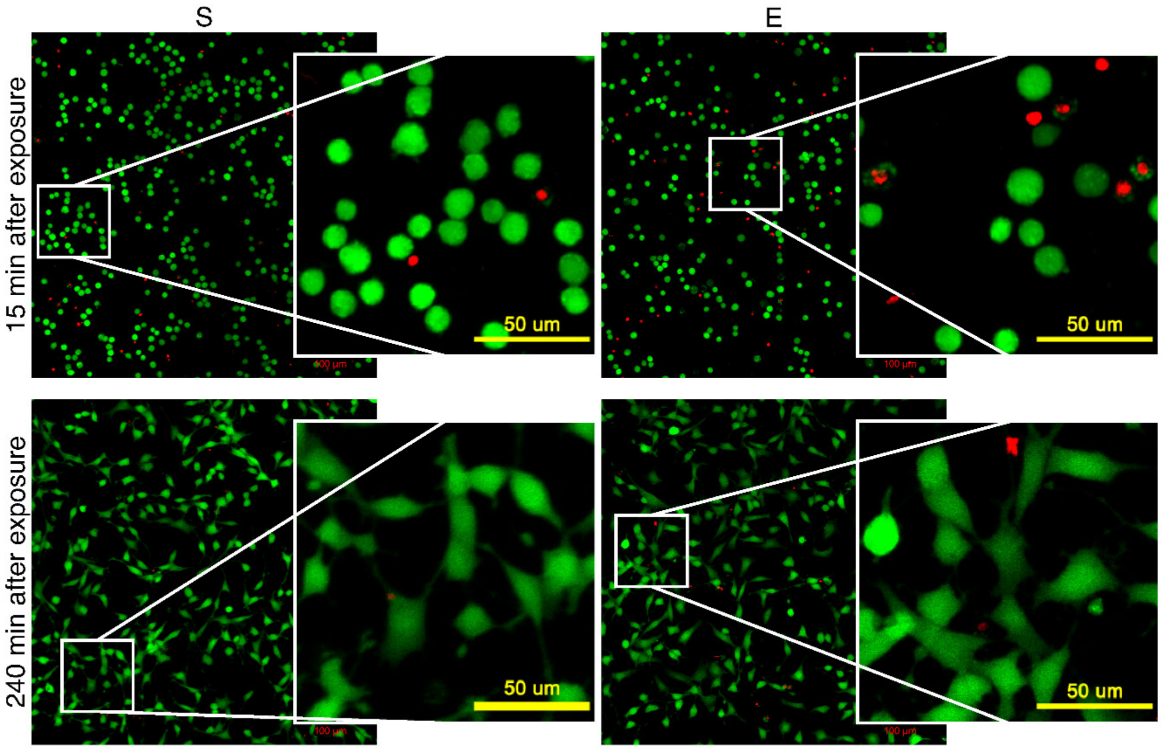

Figure S2. LIVE/DEAD™ Viability/Cytotoxicity Kit. S – sham-exposed cells, E – nsPEF-exposed cells. Images of TM3 cells stained using LIVE/DEAD™ Viability/Cytotoxicity Kit. The live cells with intact plasma membrane are green; the red colour of cells indicates the loss of integrity of the cell membrane. Images were taken 15 min (upper row) and 240 min (lower row) after nsPEF exposure. The adhesion of TM3 cells to the well's surface and typical morphology of adherent cells can be observed after 240 min of incubation.

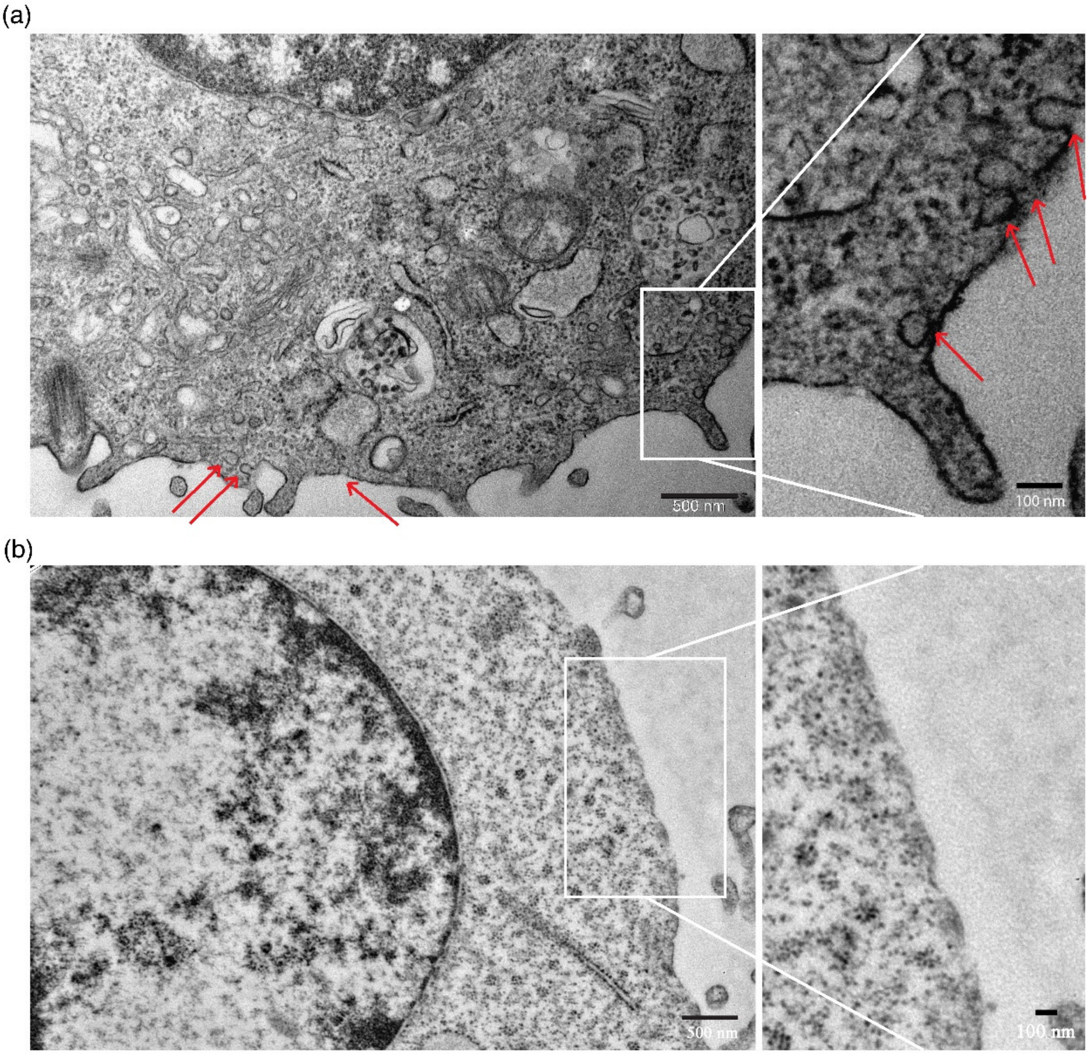

Figure S3. Structures of the cytoplasmic membrane - caveolae (red arrows) and microvilli, observed on surface TM3 cells. (**a**) Sham control. (**b**) nsPEF-exposed cells. A reduction in the number of microvilli and lack of caveolae were observed. The black bar represents 500 and 100 nm, respectively.

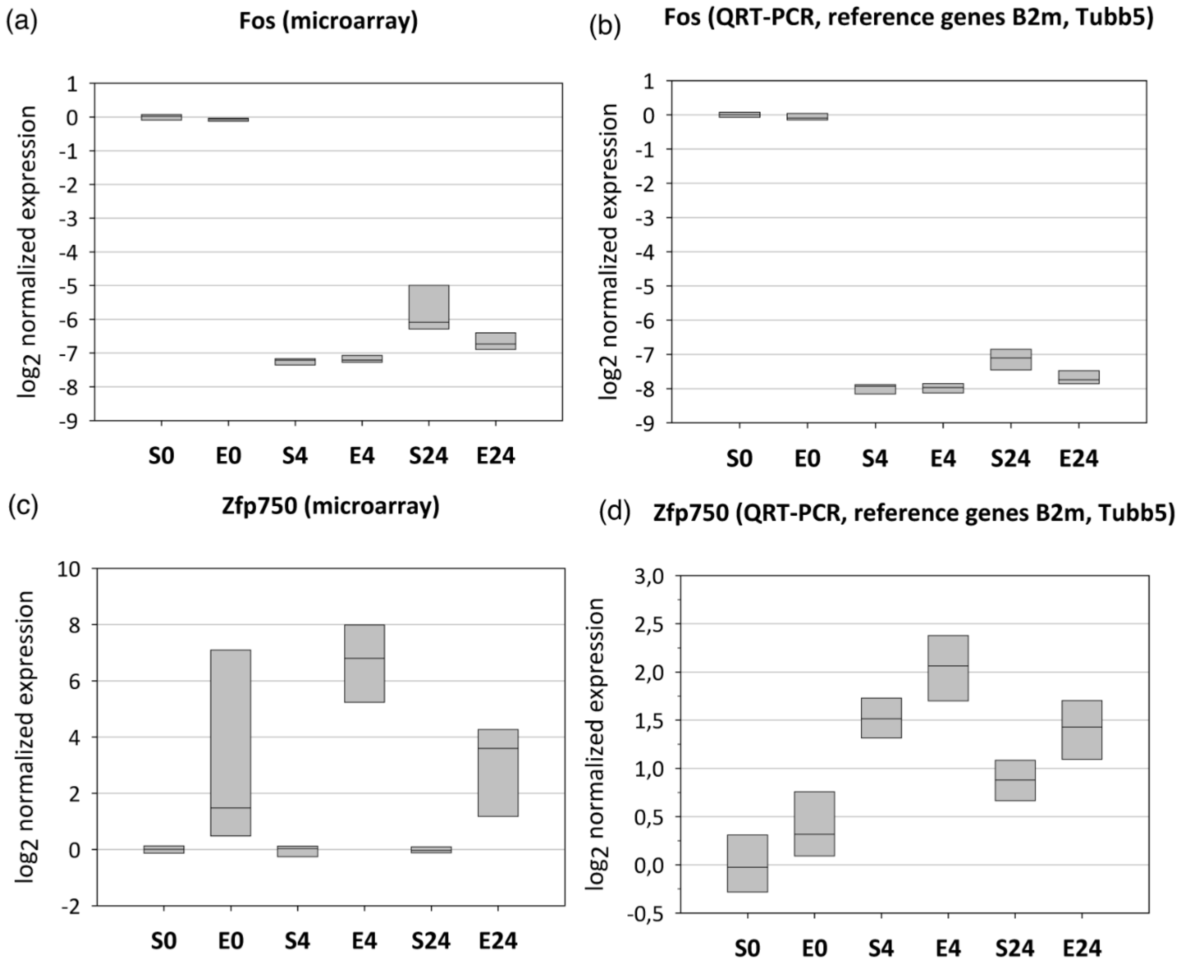

Figure S4. Confirmation of microarray results with QRT-PCR. S0 – sham-exposed cells, time 0 h; E0 – nsPEF-exposed cells, time 0 h; S4 – sham-exposed cells, time 4 h; E4 – nsPEF-exposed cells, time 4 h; S24 – sham-exposed cells, time 24 h; E24 – nsPEF-exposed cells, time 24 h. Changes in the expression of *Fos* (**a**,**b**) and *Zfp750* (**c**,**d**) in nsPEF-exposed and sham-treated TM3 cells at 0 h, 4 h or 24 h after exposure. (a, c) Gene expression was determined using microarrays. (b.d) Gene expression was determined using one step QRT-PCR with gene-specific primers (4 biological replicates for each condition). Results were calculated using ΔΔCq method and presented as log2 of relative gene expression. *B2m* and *Tubb5* were selected as endogenous controls based on their stable expression across samples. The geometric mean of the endogenous control gene expression was used as a normalization factor.
